# RFWD2 Mitigates AD‐Like Cognitive Impairments via the JNK–SGK1 Signaling Pathway in Mice

**DOI:** 10.1002/cns.70860

**Published:** 2026-04-09

**Authors:** Mengjiao Ying, Xiaochuan Qi, Ao Wang, Guangshang Zhong, Wenhui Tong, Danting Yu, Gaofeng Liu, Yu Guo

**Affiliations:** ^1^ Anhui Provincial Center for Neural Regeneration Technology and New Medical Materials Engineering Research, Bengbu Medical University Bengbu Anhui China; ^2^ School of Life Sciences, Bengbu Medical University Bengbu Anhui China; ^3^ School of Laboratory Medicine, Bengbu Medical University Bengbu Anhui China; ^4^ Affiliated Hospital of Shandong University of Traditional Chinese Medicine, Pathology Jinan Shandong China

**Keywords:** Alzheimer disease, cognitive function, JNK pathway, RFWD2, SGK1

## Abstract

**Background:**

Alzheimer disease (AD) is a degenerative disorder of the central nervous system. Its main pathological feature is the formation of neurofibrillary tangles through abnormal β‐amyloid protein (Aβ) aggregation and excessive Tau protein phosphorylation. Ring finger and WD repeating domain 2 (RFWD2) is an E3 ubiquitin ligase that regulates neuronal dendrite complexity through the c‐Jun N‐terminal kinase (JNK) pathway. This study aimed to investigate the regulatory effect of RFWD2 on the downstream protein, serum/glucocorticoid‐regulated kinase 1 (SGK1), through the JNK pathway and explore its influence on AD pathogenesis.

**Methods:**

Cognitive‐level behavioral detection was performed in RFWD2^+/−^ mice. Cultured PC12 cells and cortical neurons were also used to analyze the changes in signaling pathways caused by the decreased expression of RFWD2 in vitro and correlations between the expression of related proteins and key signaling pathways of AD at the molecular level.

**Results:**

Decreased RFWD2 expression led to cognitive deficits in AD mice, resulting in mitochondrial swelling, fragmentation of hippocampal neurons, abnormally high reactive oxygen species levels, and an imbalance between antiapoptotic and proapoptotic proteins. This effect was significantly improved by inhibiting the JNK pathway and SGK1 protein expression. Furthermore, in vitro experiments showed that in PC12 cells and cortical neurons downregulated by RFWD2, the expression levels of p‐JNK, SGK1, and p‐Tau increased, and those of LC3B/Beclin‐1 decreased; ROS levels increased, and apoptosis was induced; inhibiting JNK or SGK1 expression reversed these changes.

**Conclusion:**

RFWD2 regulates SGK1 expression through the JNK pathway, thereby regulating mitochondrial autophagy and apoptosis, altering the expression levels of p‐Tau and Aβ proteins, inducing AD‐like symptoms in mice, and promoting AD development. The RFWD2–JNK–SGK1 axis provides a valuable basis for studying the mechanisms of AD occurrence and developing early intervention strategies.

## Graphical abstract

1. The reduction in RFWD2 expression activated the JNK pathway, upregulated the levels of proteins SGK1 and p‐Tau, causing mitochondrial damage in neurons, abnormal autophagy, and driving mice to develop AD‐like pathological changes.
2. Inhibiting the JNK pathway or the expression of SGK1 can reverse the above pathological changes caused by the downregulation of RFWD2.

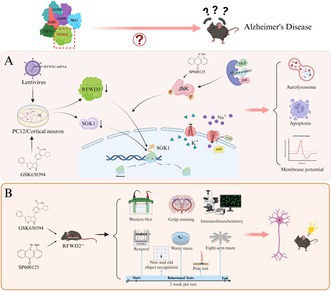

**Abbreviations**
AD: Alzheimer disease
Co‐IP: co‐immunoprecipitation
DAPI: 4′,6‐diamidino‐2‐phenylindole
JNK: c‐Jun N‐terminal kinase
LAMP2: lysosome‐associated membrane protein 2
MAPK: mitogen‐activated protein kinase
NCoR1: nuclear receptor corepressor 1
NRI: number of exploration recognition index
PBS: phosphate‐buffered saline
p‐JNK: phosphorylated c‐Jun N‐terminal kinase
RFWD2: ring finger and WD 2
ROS: reactive oxygen species
RT‐qPCR: quantitative reverse‐transcription PCR
SAP: spontaneous alternation percentage
SGK1: serum/glucocorticoid‐regulated kinase 1

## 1 Introduction

Alzheimer disease (AD) is an incurable neurodegenerative disorder that affects approximately 50 million patients worldwide. Its main characteristics include memory decline and decreased logical reasoning ability, among others [1]. The pathogenesis of AD has always been a core focus that urgently needs further investigation. From a pathological perspective, β‐amyloid protein (Aβ) in the forebrain and other key brain regions, as well as excessive phosphorylation and aggregation of the Tau protein, jointly promote the entanglement of nerve fibers and loss of nerve synapses, which are the main causes of AD onset in patients at the present stage [2]. The pathogenesis of AD is extremely complex, and mitochondrial dysfunction and oxidative stress play crucial roles in its occurrence and development [3]. Many abnormal mitochondria have been found in the brain tissues of patients with AD. For example, damaged mitochondria produce less ATP, whereas the production efficiency of reactive oxygen species (ROS) is significantly increased. Moreover, the membrane potential of damaged mitochondria in the body abnormally fluctuates, and E3 ubiquitin ligases cannot be effectively recruited to the mitochondria to label damaged proteins, thereby preventing the prompt elimination of damaged mitochondria through autophagy and causing brain dysfunction [4].

Early screening and drug treatment are two core tasks in the prevention and treatment of AD. Effective drug targets are often closely linked to molecular markers to help alleviate or cure the disease. Considering the key role E3 ubiquitin ligases play in regulating mitochondrial function and influencing AD pathogenesis, the scientific community has gradually realized the importance of novel molecules in impeding the occurrence and development of AD. Some studies have shown that microglia with ring finger and WD repeating domain 2 (RFWD2) deficiency can significantly accelerate the Tau‐mediated neurodegenerative process and interfere with the activation and conduction of inflammatory signaling pathways, as though they are adding fuel to the “flame” of neuroinflammation, thereby indirectly participating in the pathological process of AD^5^.

In mammalian cells, RFWD2 is mainly distributed in the nucleus, whereas only a small amount is present in the cytoplasm; it plays a crucial role in cell proliferation, apoptosis, and DNA repair [5, 6]. Some studies have shown that RFWD2 is highly expressed in cortical neurons and the hippocampus, is closely linked to neuronal morphology and neurogenesis, and plays an important role in neurological diseases [7]. Other studies have shown that RFWD2 can also affect neuronal dendrite complexity through the c‐Jun N‐terminal kinase (JNK) pathway and cause experimental animals to exhibit altered behavioral characteristics, such as cognitive impairments similar to those observed in AD [8]. JNK, a member of the mitogen‐activated protein kinase (MAPK) family, actively participates in various cellular responses.

To date, JNK has been identified as a subtype encoded by the three genes, JNK1, JNK2, and JNK3, and more than 10 different splicing variants have been detected [9]. JNK2 was identified based on the amino acid differences in its kinase domain [10]. JNK3 and JNK1 are similar; however, the upstream initiation site of JNK3 has an N‐terminal extension for specific expression [9]. Overall, JNK1 and JNK2 are expressed throughout the body, whereas JNK3 expression occurs only in the brain, heart, pancreatic beta cells, and testes [11]. In the nervous system, neurogenesis is delayed by the lack of JNK2 and JNK3 present but is not significantly related to JNK1, which is involved in the elongation of preformed neurites [12, 13]. In addition, JNK1 and JNK2 regulate axon regeneration by regulating the phosphorylation of microtubule‐associated protein 1 and inhibiting the increase in nerve number caused by JNK1 in the mouse hippocampal subgranular zone. In contrast, JNK3 is responsible for regulating the formation and remodeling of neurons and plays an important role in their regeneration and differentiation [14]. The activated JNK pathway can increase ROS levels, induce mitochondrial dysfunction that then results in decreased ATP levels, or affect the mitochondrial membrane potential and formation of permeability transition pores to induce apoptosis [15, 16].

In the regulatory network of long‐term memory formation, the expression of nuclear receptor corepressor 1 (NCoR1) is regulated by the JNK signaling pathway, and NCoR1 can inhibit the protein expression of serum/glucocorticoid‐regulated kinase 1 (SGK1) through CCAAT‐enhancer‐binding protein alpha. Moreover, knocking out SGK1 impairs spatial learning and memory, indicating that SGK1 is involved in long‐term memory formation [17]. JNK and SGK1 jointly regulate AD progression via their effects on cellular stress responses and neuroprotection. Synergy between the two proteins has a crucial impact on the modulation of cognitive function and pathological processes related to AD.

SGK1 plays a key role as a cellular health guard in maintaining mitochondrial function and cell health, and it plays a regulatory role in various physiological processes, such as cell growth and neuronal excitability [18]. SGK1 is highly expressed in hippocampal and cortical neurons, is involved in various processes, such as cellular ion transport and the autophagy of damaged mitochondria, and is related to the pathophysiology of AD [19, 20]. Inhibiting SGK1 can also increase the activity of glial cells to alter neuronal excitotoxicity and prevent mitochondrial damage [21]. These findings highlight the core role SGK1 plays in AD pathogenesis and highlight its potential as a key target in AD treatment.

Although notable progress has been made in AD pathogenesis research in recent years, interactions and regulatory mechanisms of the specific RFWD2–JNK–SGK1 signaling axis during the occurrence and development of AD have not been directly clarified. Therefore, this study sought to explore how decreased RFWD2 expression affects cognitive function through the JNK–SGK1 signaling pathway by constructing an RFWD2 knockdown mouse model, as well as the potential molecular mechanisms and signal transduction pathways underlying this process. The aim of this study was to elucidate the core position and key role of the RFWD2–JNK–SGK1 axis in the pathogenesis of AD and provide a solid theoretical basis and experimental foundation for the development of new AD treatment strategies based on this signaling axis.

## 2 Methods

### 2.1 Experimental Animals

Healthy C57BL/6J male and female mice were provided by Hangzhou Ziyuan Laboratory Animal Technology Co. Ltd. (Hangzhou, China) [license no. SCXK (Zhe) 2019–0004]. APP/PS1 transgenic AD model mice and the RFWD2^+−−^ female and male mice that had been bred from gene knockouts of C57BL/6J wild‐type mice were supplied by Saiye (Suzhou) Model Biology Research Center Co. Ltd. (Suzhou, China) (RFWD2 homozygous knockout embryo lethal) [License No. SCXK (Su) 2018–0003]. The animals were raised in a clean‐grade animal room, with the environmental temperature controlled at 23°C ± 1°C, relative humidity at 55%–65%, and light–dark cycle of 12 h each. Experimental procedures were performed in accordance with the requirements of the Guidelines for the Review of Laboratory Animal Welfare Ethics (GB/T 35892‐2018) in China, and the welfare ethics of laboratory animals implemented in a standardized manner. All experiments were designed and reported in accordance with the Animal Research: Reporting of In Vivo Experiments (ARRIVE) guidelines. The use of experimental animals and all experimental procedures were approved by the Ethics Committee of Bengbu Medical College (Bengbu, China) (Approval No. 2024‐565).

### 2.2 Main Reagents

The following is a list of all key reagents used in this study: DMEM basic (Gibco, Waltham, MA, USA), neurobasal medium [−] l‐glutamine (Gibco), B‐27 serum‐free supplement (Gibco), GlutaMAX (Gibco), fetal bovine serum (Gibco), antibiotic‐antimycotic (Gibco), 0.25% EDTA‐containing phenol red trypsin (1 ×) (Gibco), poly‐d‐lysine (PDL) (Sigma‐Aldrich, St. Louis, MO, USA), SP600125 (MedChemExpress, Monmouth Junction, NJ, USA), GSK650394 (MedChemExpress), phosphatase inhibitor cocktail (Bioss, Woburn, MA, USA), RNA Extraction Kit (Vazyme, Nanjing, China), TUNEL (Cat. No: HY‐K1078; MedChemExpress), 2′, 7′‐dichlorodihydrofluorescein diacetate fluorescent probe (Cat: HY‐D0940; MedChemExpress), Annexin‐V‐FITC (Cat. No: HY‐K1073; MedChemExpress), Mitochondrial Membrane Potential Detection Kit (Beyotime Biotechnology, Shanghai, China), mouse monoclonal anti‐RFWD2 (Sigma‐Aldrich), rabbit polyclonal anti‐SGK1 (Wuhan Proteintech, Wuhan China), rabbit polyclonal anti‐phosphorylated JNK (*p*‐JNK) (Santa Cruz Biotechnology, Dallas, TX, USA), mouse polyclonal anti‐p‐Tau (Santa Cruz Biotechnology), rabbit polyclonal anti‐Kv1.4 (Invitrogen, Waltham, MA, USA), mouse monoclonal anti‐MAP‐2 (Santa Cruz Biotechnology), mouse polyclonal anti‐p‐JNK (Santa Cruz Biotechnology), rabbit polyclonal anti‐JNK3 (Abcam, Cambridge, UK), donkey anti‐rabbit Cy3 (The Jackson Laboratory, Bar Harbor, ME, USA), donkey anti‐mouse Alexa Fluor 488 (Invitrogen), Anti‐Fluorescence Quenching Mounting Medium (Invitrogen), PAGE Gel Rapid Preparation Kit (Yeasen Biotechnology, Shanghai, China), and bovine serum albumin (full component) (Solarbio, Beijing, China).

### 2.3 Lentiviral Construction and Transfection

The gene sequence (CTCAACTCCTACGAGGACAAA) of the previously verified effective RFWD2‐specific shRNA was obtained from the NCBI website, and the LV‐RFWD2‐shRNA‐mCherry lentivirus was constructed by GenePharma (Suzhou, China). The obtained lentivirus titer was 1 × 10^8^ TU/mL, and the lentivirus was stored at −80°C until use.

PC12 cells (rat adrenal pheochromocytoma cells) were seeded at a density of 5000 cells/well in 24‐well plates and divided into four groups. The blank control group (Ctrl group) received no treatment, and the medium was refreshed normally. The other three groups included the RFWD2‐shRNA, RFWD2‐shRNA + GSK, and RFWD2‐shRNA + SP groups. When the cells reached approximately 70% confluence, they were transfected with LV‐RFWD2‐shRNA‐mCherry (MOI = 70) for 48 h, and a final concentration of 5 μg/mL of the transfection aid (polybrene) was added to enhance viral infection. The cells were treated with 2 μg/mL puromycin for 48 h for screening. When all control cells were killed by puromycin, cells in the virus group that had survived and whose fluorescence could be observed were considered positive cells successfully infected by the virus, thus representing the RFWD2 knockdown cell line. The expression levels of RFWD2 in each group were evaluated through western blotting analysis and quantitative reverse‐transcription PCR (RT‐qPCR) (primer sequences are shown in Table 1). After generating cells with low RFWD2 expression via lentivirus infection, the RFWD2‐shRNA + GSK and RFWD2‐shRNA + SP groups were treated with the JNK inhibitor, SP600125 (10 μM, 1 h), and the SGK1 inhibitor, GSK650394 (20 μM, 24 h), respectively, whereafter they were subjected to western blotting and immunofluorescence analyses. The cells were treated with MG132 for proteasome inhibition and transfected with plasmids overexpressing JNK with His tags. Subsequently, ubiquitination levels were detected via western blotting [22].

**TABLE 1:** Primer sequences used in this study.

Name	Sequence (5′ → 3′)
RFWD2—Homozygous 706—F1	TTTATTCCACTCTTACTTGGCCTTC
RFWD2—Homozygous 706—R1	CAAGAGCAGAGAATTACCTTCACG
RFWD2—Heterozygous 469—F2	CTTGTTTTAGTGTCCTGAGTGCTG
RFWD2‐F	TGGCCACAGCTTTTGCTACA
RFWD2‐R	TGCCACCTATGCCCATTTGA
β‐Actin‐F	TTCTACAATGAGCTGCGTGTG
β‐Actin‐R	GGGGTGTTGAAGGTCTCAA

Mouse embryonic cerebral cortical neurons at embryonic day 16 were collected and seeded at 0.12 × 10^6^/well onto PDL‐coated coverslips. On the 9th day of culture, the cerebral cortical neurons were divided into four groups. The medium of the Ctrl group was changed to neurobasal medium (supplemented with 2% B27 + A, 0.25% GlutaMAX, and 1% triple antibiotics). The other three groups (RFWD2‐shRNA, RFWD2‐shRNA + GSK, and RFWD2‐shRNA + SP) were subjected to lentivirus infection. When the medium was refreshed, half was removed and labeled as the old medium for later use. This was mixed with fresh medium containing the LV‐RFWD2‐shRNA‐mCherry lentivirus (MOI = 70), but without polybrene, to transfect the cortical neurons. After 48 h, the medium was replaced with fresh medium for continued culture. On the 12th day, cortical neurons in the RFWD2‐shRNA + GSK group were treated with GSK650394 at a final concentration of 20 μM for 24 h. Cortical neurons of the RFWD2‐shRNA + SP group were treated with SP600125 at a final concentration of 10 μM for 1 h on the 13th day. After all treatments, proteins were extracted from the four groups for western blotting and immunofluorescence analyses.

### 2.4 Transcriptome Sequencing

Using the public functional genomics database, Gene Expression Omnibus, the keyword “Alzheimer's disease” was searched, and the GSE269222 series was used as the analysis dataset. This study included significantly differentially expressed gene (DEG) sequences between normal and AD model mice. The “heatmap,” “limma,” and “edgeR” packages of R software were used to draw heatmaps of the DEGs, and GO and KEGG enrichment analyses were performed via the gene annotation and analysis resource website (http://www.bioinformatics.com.cn/).

Based on the sequence of the mural‐derived gene, MIR155 (Cop1)*2–2, in NCBI GenBank, four sequences targeting mouse RFWD2 were designed. These sequences have been proven effective in a previous study. Using GV475 as the vector, which carried the hSyn promoter‐mcs‐cherry‐3flag‐sv40 polyA, four interference sequences were inserted into the T2A‐isolated vector to successfully construct the shRNA‐carrying adeno‐associated virus (AAV9‐hSyn‐RFWD2‐shRNA‐mCherry) (Table 2). Male C57BL/6J mice, aged 8 weeks and weighing 22–24 g, were anesthetized with isoflurane gas. The experimental mice were further divided into three groups. The NC group received a 0.45‐μL normal saline injection into the bilateral mPFC area (A/*P* + 1.70 mm, M/L ±0.40 mm, D/V‐1.80 mm) at a rate of 0.045 μL/min using a stereotactic brain, according to the mouse brain atlas. The Vehicle and RFWD2‐shRNA groups were injected with empty viruses or AAV‐RFWD2‐shRNA, respectively, into the bilateral mPFC areas at the same speed as that of the NC group. To further determine the effect of RFWD2 expression on the gene expression profile of mice, at least 0.5 μg total RNA was isolated from each sample [three samples from the wild‐type (W), adeno‐associated virus (A), and RFWD2 knockout (R) groups each; total of nine samples] using TRIzol reagent (Invitrogen) and analyzed with a Nanodrop (NanoDrop Technologies, Wilmington, DE, USA) and Agilent 2100 system (Agilent Technologies, San Diego, CA, USA). Sequencing was performed on the DNBSEQ‐T7 platform of Wuhan Frasergen Bioinformatics Technology Co. Ltd. (Wuhan, China).

**TABLE 2:** RFWD2‐shRNA sequences.

Number	Sequence (5′ → 3′)
RFWD2 shRNA1	GCTGGAGTTACAAAGAAGATT
RFWD2 shRNA2	CTACAAGGATGTCTCGTAT
RFWD2 shRNA3	AAGTGTATTCATCAGAGTTTGGA
RFWD2 shRNA4	CTCAACTCCTACGAGGACAAA

### 2.5 Differentially Expressed Genes and Their Functional Enrichment

SOAPnuke (v.2.1.0) was used to filter the sequencing data to remove low‐quality reads (reads that had a number of bases with a Qphred score ≤ 20 accounting for > 50% of the entire read length). Clean reads were then obtained. Using RSEM (v.1.3.1), the alignment results of Bowtie2 (v.2.3.5) were called for statistics to obtain the number of reads mapped to each transcript in each sample and perform FPKM conversion. Genes with or without biological replicates were subjected to significant differential expression analysis using DESeq2 (v.1.22.2) or edgeR (v.3.6.8), respectively, and the screening thresholds used included a false discovery rate < 0.05 and log_2_FC (fold change) > 1 or < −1. KEGG enrichment analysis was performed on the DEGs.

### 2.6 Behavioral Tests

The RFWD2^+/−^ mice were divided into four groups: the wild‐type (Wild‐Type; *n* = 10), RFWD2^+/−^ (shRNA‐RFWD2; *n* = 10), RFWD2^+/−^ + SP600125 (*n* = 10; intraperitoneally injected with 10 mg/kg SP600125 for 10 consecutive days), and RFWD2^+/−^ + GSK650394 groups (shRNA‐RFWD2^+/−^ GSK650394, *n* = 10; intraperitoneally injected with 30 mg/kg GSK650394 for eight consecutive days). Each behavioral test was separated by one week and conducted in the same quiet environment.

New object recognition test: This test consisted of two phases. In the first stage, two identical objects, A and B, were placed in an open field box 12.5 cm from the wall of the box, and the experimental mice were allowed to explore the old object freely for 10 min. After the first phase of the experiment, the mice were allowed to rest for 1 h before the second phase of the experiment commenced. Object A was replaced inside the box with object C, which had a different shape, and the same operation was then performed as that during the first stage. The ratio of the number of times a mouse explored a new object to the number of times it explored an old object was called the number of exploration recognition index (NRI).

Water maze: The maze used had a circular pool structure, the water temperature was maintained at approximately 26°C, and a space marker was present. The adaptation period was set to one day, and an escape platform was set 1 cm above the water surface, with clear and transparent water, designed to allow experimental mice to explore and remember the location of the platform. The platform was then moved below the surface, and titanium dioxide was added to muddy the water, after which the platform entered the training phase for six days. On day 8, a 3‐day formal test began, in which the trajectory of the mouse looking for the platform and the time it took were recorded. Each experimental animal was tested three times, and the time interval between two adjacent tests remained above 2 min.

Y‐maze: The Y‐maze was divided into two sections for detection purposes. Spontaneous alternation behavior tests were conducted on days 1–3 to evaluate the spatial working memory of the mice. The mice were allowed to rest for 4–8 days, whereafter spatial recognition memory tests were conducted on days 9–13 to evaluate their long‐term spatial memory.

Spontaneous alternation behavior test: The maze used was Y‐shaped and comprised three arms of equal length. Each arm was 10 cm wide and 40 cm long, with a 120° angle between them. On the first and second days, the mice were placed in the Y‐maze for 8 min of free exploration. To familiarize the mice with the structure and smell of the Y‐maze, they were placed in the central area of the Y‐maze (where the three arms meet) on the third day, facing one of the arms (the starting arm), and then allowed to freely explore the maze for 10 min. The total number of times mice entered the arms (denoted as N) and spontaneous alternation rate (denoted as SA, that is, the number of consecutive entries into different arms three times) were recorded. The spontaneous alternation percentage (SAP) was calculated via the following formula: SAP (%) = (SA/(*N* − 2)) × 100.

Spatial recognition memory test: Rewards were placed in the target arm on days 1–3. Mice were placed in the starting arm and allowed to explore the maze. Two trials were conducted each day at 1 h intervals. On days 4–5, the rewards in the target arm were removed, and the mice were placed in the starting arm again for free exploration. The number of entries into the target arm, duration of stay, and transitions between various arms were recorded.

### 2.7 Western Blotting

Cells and brain tissues from the Wild‐Type, RFWD2‐shRNA, RFWD2‐shRNA + SP, and RFWD2‐shRNA + GSK groups were extracted using a total protein extraction kit, according to the manufacturer's instructions (cat. no. P0028; Beyotime Biotechnology). Equal amounts of protein from each sample were separated via 10% SDS‐PAGE, transferred to polyvinylidene fluoride membranes (0.45 μM; Millipore, Burlington, MA, USA), and immunoblotted overnight at 4°C with primary antibodies. On the second day, the membranes were incubated with an IgG‐HRP antibody (dilution ratio of 1:5000) at room temperature for 1 h, visualized with Clarity Western ECL Blotting Substrates (Bio‐Rad Laboratories, Hercules, CA, USA), and analyzed using Bio‐Rad Image Lab software (Bio‐Rad Laboratories).

### 2.8 Immunofluorescence

Coverslips containing cells and brain tissue sections were fixed with 4% paraformaldehyde, permeabilized with 0.2% Triton X‐100 for 10 min, and blocked with 2% bovine serum albumin and 10% normal donkey serum for 1 h at room temperature. The blocking solution was subsequently washed away and incubated with rabbit polyclonal anti‐RFWD2 (1:200; Bioss), rabbit polyclonal anti‐SGK1, rabbit polyclonal anti‐p‐JNK, mouse polyclonal anti‐p‐Tau, rabbit polyclonal anti‐Kv1.4, and mouse monoclonal anti‐MAP‐2 antibodies at 4°C overnight. After the primary antibodies were removed through washing with phosphate‐buffered saline (PBS), the corresponding secondary antibodies, Alexa Fluor 488 (1:500) and Alexa Fluor Cy3 (1:1000), were added, and the samples incubated for 1 h. DAPI was then used for counterstaining, and an anti‐fluorescence quenching agent was used to seal the slides. Observations were made using a confocal microscope (FV1200MPE SHARE; Olympus, Tokyo, Japan).

### 2.9 Transmission Electron Microscopy

When PC12 cell cultures reached 70%–80% confluence, they were washed once with PBS, whereafter the cell pellet was collected via centrifugation. The cell pellet was fixed with 2.5% glutaraldehyde for 2 h, followed by three rinses with 0.1 M phosphate rinse solution for 15 min each and fixation with 1% osmium acid fixative for 2–3 h; then, the same procedure was performed with 0.1 M phosphate rinse solution. Afterwards, dehydration treatment was carried out. Briefly, a 30% dehydrating agent was prepared by mixing acetone and sterilized water at a volume ratio of 3:7. PBS in the sample tube was aspirated and discarded, and the freshly prepared dehydrating agent quickly added, and the sample left stationary at room temperature for 45 min. Dehydration was carried out at concentrations of 30%, 50%, 70%, 90%, and 100% (v/v). The embedding treatment was carried out as follows: After mixing pure acetone + embedding solution (2:1), the mixture was incubated at room temperature for 3–4 h, the pure acetone + embedding solution (1:2) incubated at room temperature overnight, and the pure embedding solution incubated at 37°C for 2–3 h. The samples were subsequently heated at 37°C, 45°C, and 60°C for 12 h, 12 h, and 24 h, respectively, to achieve curing. The embedding blocks were cut into 50–60‐nm thin sections via a Lecia ultramicrotome (EMUC7; Leica, Wetzlar, Germany), double‐stained with 3% uranyl acetate–lead citrate, and the sections finally observed under a transmission electron microscope (JEM‐1400; JEOL, Tokyo, Japan).

### 2.10 Statistical Analysis

The graphical summaries and behavioral flowcharts were all drawn on the Biorender website and obtained permission and copyright (Figures S3 and S4, Number: PN281PVA13, MD281PV7UO). All statistical analyses were performed using GraphPad Prism v.9.0 (GraphPad Software, San Diego, CA, USA). The experimental data are presented as the means ± standard errors of the means. One‐way or two‐way analysis of variance or two‐tailed unpaired *t*‐tests were used to calculate *p*‐values, with *p* < 0.05 considered statistically significant.

## 3 Results

### 3.1 Sequencing Data Analysis of AD Model Mice

**FIGURE 1:**
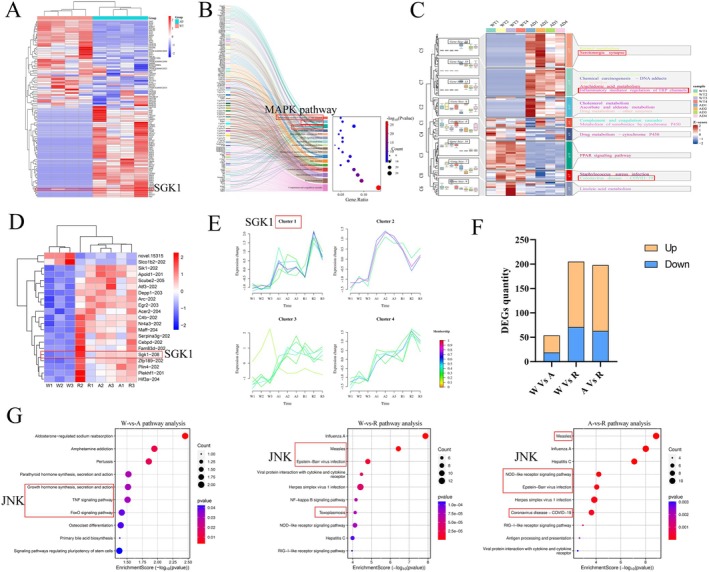
Analysis of Differentially Expressed Genes in the AD Database and Pathway Analysis Diagram of Transcriptome Sequencing with Reduced RFWD2 Expression. (A) Heatmap of differential gene expression in the AD database, with the significantly differentially expressed gene (DEG), *SGK1*, circled in the red box; (B) Mulberry bubble plot of the KEGG enrichment analysis of DEGs in AD, with pathways related to the MAPK pathway marked in the red box; (C) Combined diagram of the boxplot of gene expression trend changes, heatmap, and pathway clustering analysis, with the MAPK‐related pathways circled in the red box; (D) Heatmap of differential gene expression in the transcriptome sequencing analysis of groups W, A, and R, with the *SGK1* gene selected in the red box (W, Wild‐Type group; A, adeno‐associated virus group; and R, RFWD2 knockout group); (E) Bar graph of the DEG quantity in groups W, A, and R; (F) Trend line graph of the cluster analysis of DEGs in groups W, A, and R, showing the upregulated expression of *SGK1*.

By comparing the gene sequences of Wild‐Type and AD model mice sourced from the GSE269222 dataset and setting the cutoff value as |log_2_FC| > 1 and *p*‐value < 0.05, a heatmap of DEGs was generated, and the expression of SGK1 in AD model mice found to be significantly upregulated (Figure 1A). A mulberry bubble plot was generated through KEGG enrichment analysis of the DEGs, and the onset of AD found to be significantly related to the MAPK pathway (Figure 1B). A boxplot of gene expression trend changes, heatmap, and pathway clustering analyses further revealed that *SGK1*, *Card9*, *Ptpn6*, *Fgg*, and *Fga* genes were closely associated with the MAPK pathway, confirming the importance of the MAPK pathway in AD (Figure 1C).

Transcriptome sequencing analysis was performed for groups W, A, and R to obtain the PRJNA1109281 dataset. Compared with those in the W group, 54 DEGs were identified in the Agroup, with the upregulated and downregulated genes accounting for 64.81% (35 genes) and 35.19% (19 genes), respectively. Among the group W and R mice, 205 DEGs were identified, with the upregulated and downregulated genes accounting for 65.37% (134 genes) and 34.63% (71 genes), respectively. Among the group A and R mice, 198 DEGs were identified, with the upregulated and downregulated genes accounting for 68.18% (135 genes) and 31.82% (63 genes), respectively (Figure 1E) A heatmap of DEGs revealed the differential expression of *SGK1* among the different groups (Figure 1D). Cluster analysis of the DEGs yielded a trend line graph (Figure 1F), and the expression of *SGK1* was found to be significantly upregulated. Pathway cluster analysis of DEGs among the W‐A, W‐R, and A‐R groups revealed a significant correlation with the JNK signaling pathway (Figure S6A–C). In addition, by constructing APP/PS1 transgenic mice to simulate patients with AD, western blotting results showed that the protein levels of p‐JNK in APP/PS1 samples significantly increased, whereas those of RFWD2 significantly decreased (Figure S7A). Therefore, the expression of *SGK1* and the MAPK pathway may be related to the occurrence of AD. A change in expression levels of the E3 ubiquitin ligase, RFWD2, can cause changes in *SGK1* expression and is associated with the JNK pathway, which may be involved in AD.

### 3.2 Multitechnique Combination Reveals the Interaction Relationships Among RFWD2, JNK, and SGK1

**FIGURE 2:**
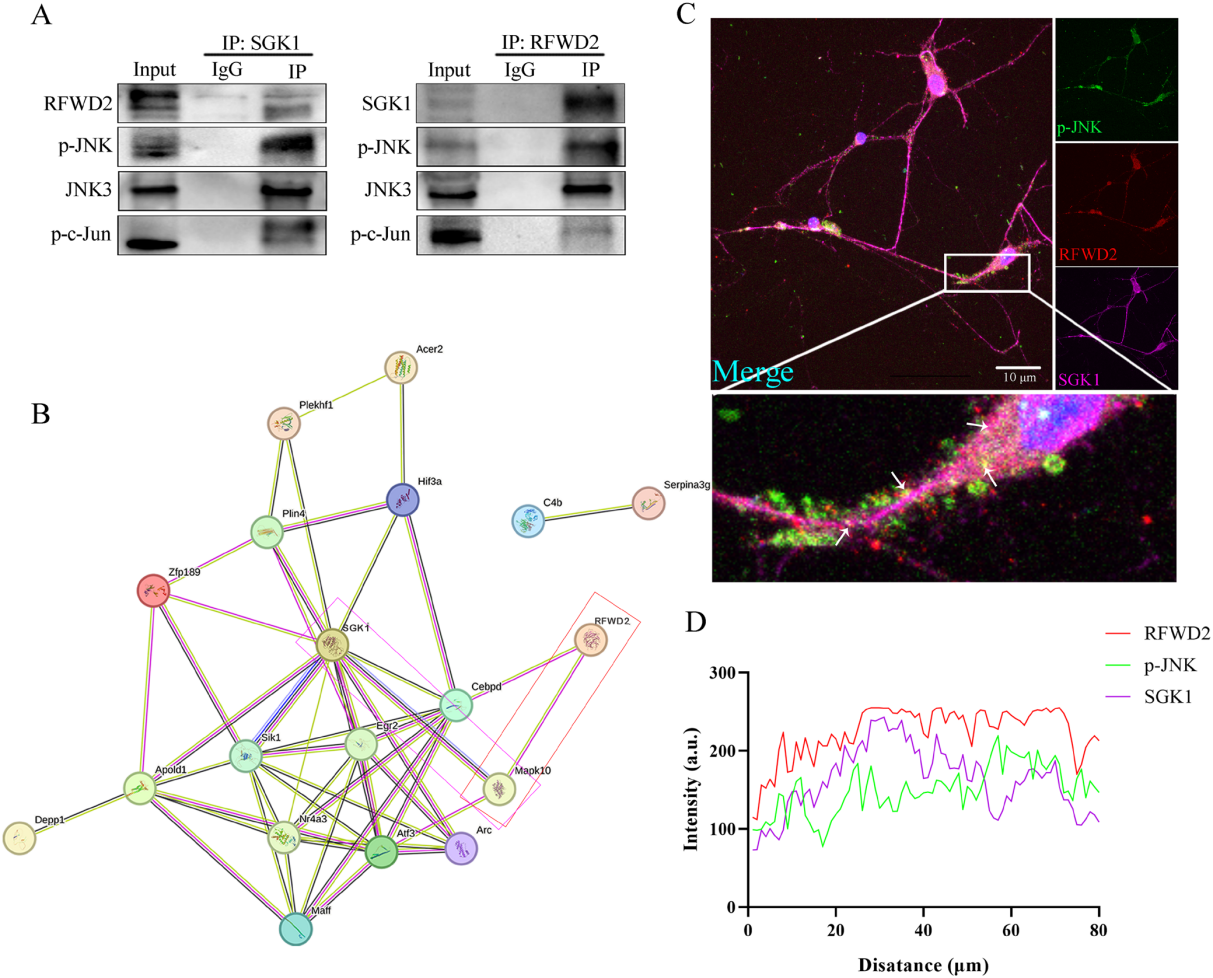
Comprehensive overview of the interaction relationships among RFWD2, JNK, and SGK1. (A) Co‐IP experimental results. The left band uses SGK1 as the bait antibody, whereas the right band uses RFWD2. (B) Bioinformatics protein interaction analysis of the differentially expressed genes obtained from the W‐A, W‐R, and A‐R groups were used to generate an interaction map between the proteins. (C) Cortical neuron immunofluorescence. Red: Cortical neurons infected with LV‐RFWD2‐shRNA‐mCherry emit red fluorescence; green: P‐JNK; purple: SGK1; blue: Nucleus. White arrows in the figure point to the overlap area of the three colocalizations. (D) Trend line graph of the Pearson correlation coefficient analysis.

Proteins were extracted from the cortical tissue of C57 neonatal mice for co‐immunoprecipitation (Co‐IP) detection. The results revealed clear interactions between RFWD2 and both JNK and SGK1 (Figure 2A). In addition, interactions were observed among RFWD2, SGK1, the potassium channel protein, Kv1.4, and Tau protein (Figure 2A). In addition, a significant interaction was observed between SGK1 and the JNK (MAPK 10) pathway, as well as an association between the JNK pathway and RFWD2 (Figure 2B). Immunofluorescence results of cortical neurons indicated that RFWD2, p‐JNK, and SGK1 were colocalized in neurons, confirming their spatial interaction (Figure 2C). Pearson correlation coefficient analysis results of fluorescence intensity revealed a correlation trend among the three genes, indicating a good correlation between RFWD2 and SGK1 (Figure 2D).

### 3.3 Downregulation of RFWD2 Activates the JNK Pathway and Upregulates the Protein Expression of SGK1

**FIGURE 3:**
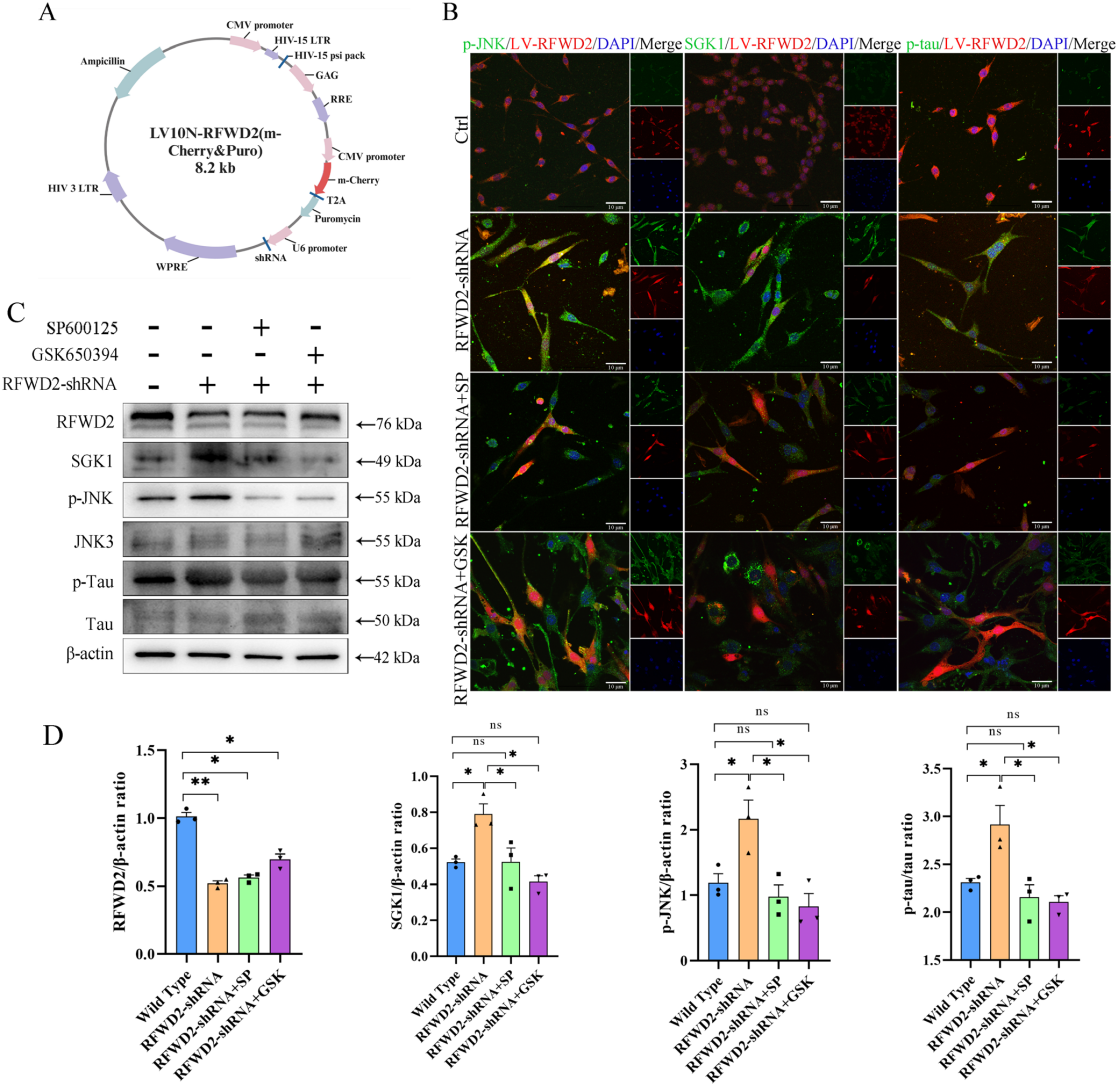
(A) Construction of the LV‐RFWD2‐shRNA lentiviruses; (B) Confocal fluorescence intensity of PC12 cells. Ctrl represents the blank control group; LV‐RFWD2 the group infected with the LV‐RFWD2‐shRNA‐mCherry lentivirus; LV‐RFWD2 + SP the group treated with SP600125 (10 μM, 1 h) after infection with the LV‐RFWD2‐mCherry; and LV‐RFWD2 + GS the group treated with GSK650394 (20 μM, 24 h) after infection with LV‐RFWD2‐mCherry. Red: Ctrl represents RFWD2, and the other groups represent LV‐RFWD2‐shRNA‐m‐Cherry; green: P‐JNK, SGK1, and p‐Tau; blue: DAPI; (original magnification: 600 ×); (C) Western blotting detection results of protein lysates prepared after cells were treated with lentiviruses, GSK650394, and SP600125; (D) Bar graph of the ratio of each western blotting target band to that of β‐Actin.

The RT‐qPCR and western blotting results showed that LV‐RFWD2‐shRNA could effectively reduce RFWD2 expression (Figure S1A–C). In addition, the Vehicle had no effect on RFWD2 expression (Figure S5A,B). RT‐qPCR results revealed that the mRNA expression levels of RFWD2 significantly decreased after LV‐RFWD2‐shRNA lentivirus infection (Figure 3A). Suitable SP600125 and GSK650394 concentrations were determined to be 10 and 20 μM, respectively, by using a cell viability assay and western blotting detection (Figure S2). After inhibiting RFWD2 expression, the expression levels of SGK1, *p*‐JNK, Kv1.4, and *p*‐Tau significantly increased (*p* < 0.05; Figure 3C,D). However, after SP600125 was used to inhibit JNK pathway activity, the expression levels of these proteins were restored, and a similar result obtained after SGK1 expression was inhibited by GSK650394 (Figure 3C,D). Confocal immunofluorescence staining revealed that in RFWD2‐downregulated PC12 cells, the expression of SGK1, *p*‐JNK, and *p*‐Tau significantly increased, whereas their expression significantly decreased after treatment with SP600125 and GSK650394 (Figure 3B), which was consistent with the western blotting results. In addition, by constructing cell models overexpressing HIS‐tagged JNK and transfecting them with the Vector or RFWD2‐shrNA, and by using MG132 to inhibit proteasome degradation, the Co‐IP results showed that after knocking down RFWD2, the ubiquitination modification levels of JNK was significantly reduced. However, no significant differences in the expression levels of His‐JNK were noted among the total cellular proteins. MG132 treatment eliminated the interference that proteasome degradation has on JNK protein levels (Figure S7B). These results indicate that RFWD2 reduction activated the JNK pathway, causing an increase in SGK1 expression and Tau protein phosphorylation.

### 3.4 Regulatory Effects of the RFWD2–JNK–SGK1 Axis on the Morphology and Function of Mitochondria in PC12 Cells

**FIGURE 4:**
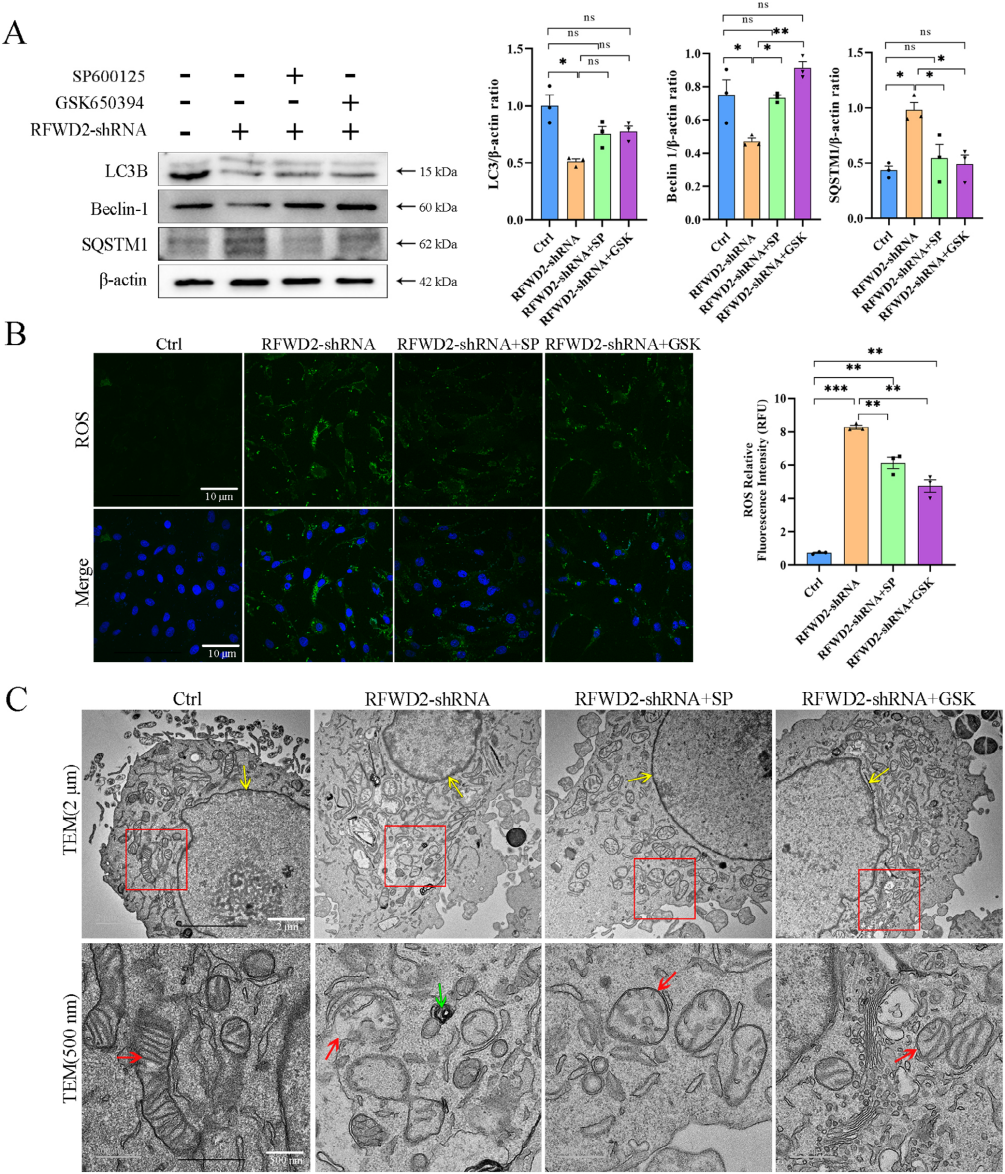
RFWD2–JNK–SGK1 axis‐mediated effects on the morphology, size, and function of mitochondria in PC12 cells. (A) PC12 cellular proteins were extracted for western blotting detection, and the righthand side shows the grayscale value bar graph analysis; (B) Detection of ROS levels. Green: ROS dye, blue: DAPI; (C) Cell transmission electron micrographs. Yellow arrow: Nuclear membrane, red arrow: Mitochondria, green arrow: Iron ion deposition.

After RFWD2 expression levels decreased, those of the autophagy‐related proteins, LC3B and Beclin‐1, significantly decreased (*p* < 0.05), whereas those of the autophagy substrate protein, SQSTM1, significantly increased (*p* < 0.05) (Figure 4A). However, when SP600125 and GSK650394 were used to inhibit JNK pathway activity and SGK1 expression, the opposite trend was observed. These findings indicate that a decrease in RFWD2 expression inhibits autophagy, whereas JNK pathway inhibition and decreased SGK1 expression lead to an increase in autophagic flux. When RFWD2 expression was inhibited, intracellular ROS levels increased, whereas they decreased after SP600125 and GSK650394 were added (Figure 4B) Compared with those in the control group, the ultrastructures of mitochondria in the RFWD2‐shRNA group were swollen, the cristae structure was blurred, the nuclear membrane was invaginated and atrophied, and electron‐dense aggregates were observed (Figure 4C). Compared with those in the RFWD2‐shRNA group, the mitochondrial morphology in the RFWD2‐shRNA + SP and RFWD2‐shRNA + GSK groups improved, mitochondrial size tended to be normal, the crista structure of some mitochondria gradually recovered, and the degree of swelling decreased (Figure 4C). However, the nuclear membrane shape in the RFWD2‐shRNA + SP group was normal, whereas that in the RFWD2‐shRNA + GSK group was irregular.

### 3.5 RFWD2–JNK–SGK1 Axis Positively Regulates Apoptosis in PC12 Cells

**FIGURE 5:**
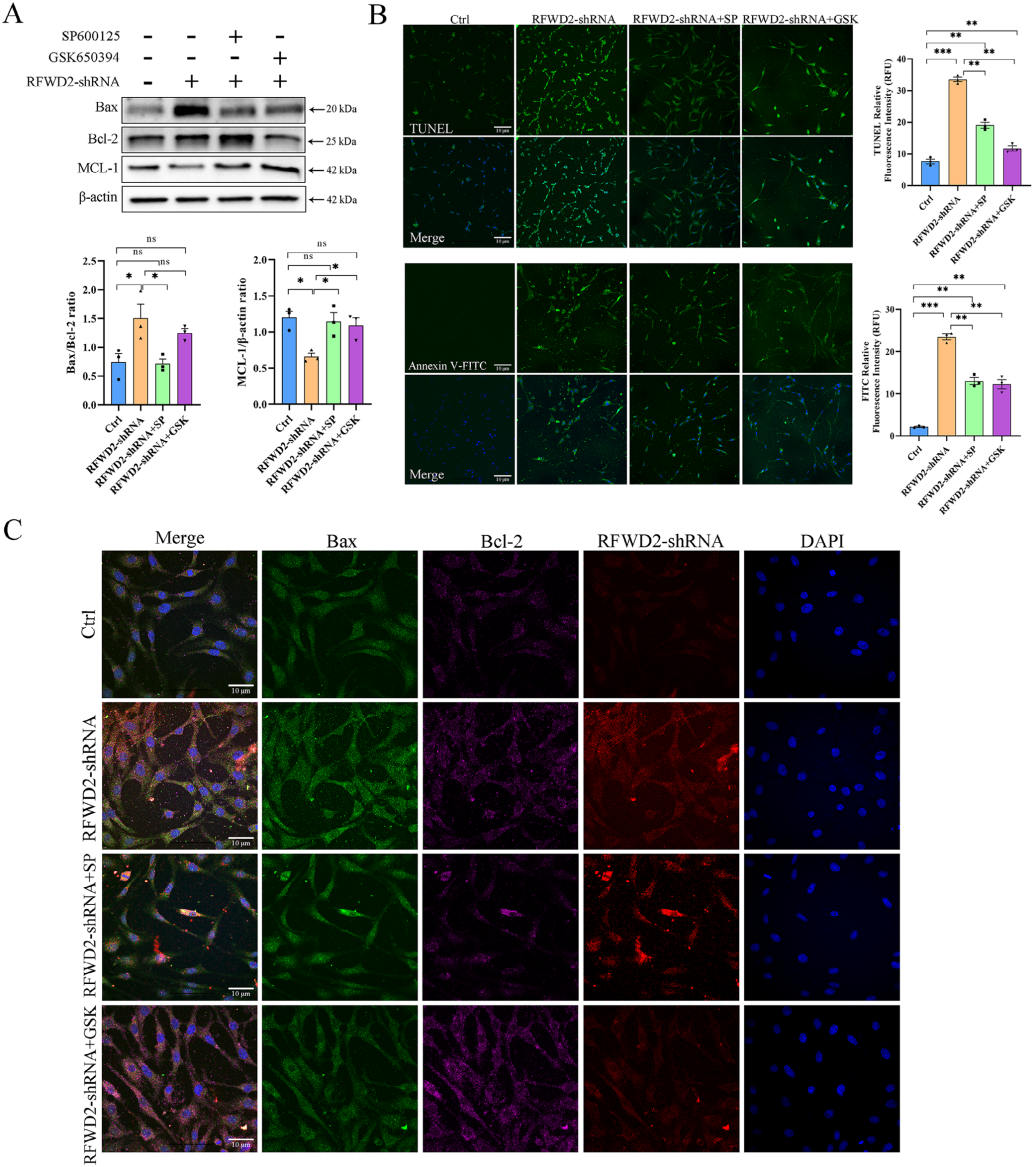
(A) Western blotting analysis of the expression of apoptosis‐related proteins in PC12 cells of different treatment groups. The bottom bar graph shows the Bax/Bcl‐2 ratio and relative expression levels of MCL‐1 in each treatment group. (B) Detection of apoptosis in PC12 cells from different treatment groups measured using TUNEL and Annexin V‐FITC staining. The green fluorescence from TUNEL staining (top) represents positive apoptotic cells, and that from Annexin V staining (bottom) represents positive cells in the early stage of apoptosis. The bar graph on the right shows the number of apoptotic cells in each treatment group. (C) Immunofluorescence staining was used to detect the expression of apoptosis‐related proteins in PC12 cells from different treatment groups. Green: Bax; purple: Bcl‐2; red: LV‐RFWD2‐shRNA‐mCherry; blue: DAPI.

In the RFWD2‐shRNA expression group, the Bax/Bcl‐2 ratio significantly increased (*p* < 0.05), whereas expression levels of the antiapoptotic protein, MCL‐1, significantly decreased (*p* < 0.05), indicating that the inhibition of RFWD2 expression could induce apoptosis (Figure 5A). Compared with that in the RFWD2‐shRNA group, the Bax/Bcl‐2 ratio in the SP600125 and GSK650394 groups was significantly lower, and the expression levels of MCL‐1 was restored. In addition, the number of apoptotic cells in the RFWD2‐shRNA group significantly increased (*p* < 0.01), whereas that in the RFWD2‐shRNA + SP and RFWD2‐shRNA + GSK groups was lower than that in the RFWD2‐shRNA group (Figure 5B). The immunofluorescence results revealed that the expression levels of Bax and Bcl‐2 were consistent with those determined via western blotting (Figure 5C). These results suggest that the RFWD2–JNK–SGK1 axis regulates apoptosis, and that inhibiting the JNK or SGK1 pathway can effectively reverse changes in the expression of apoptosis‐related proteins caused by the inhibition of RFWD2.

### 3.6 Expression and Localization of Key Proteins Related to the RFWD2–JNK–SGK1 Axis in Cortical Neurons

**FIGURE 6:**
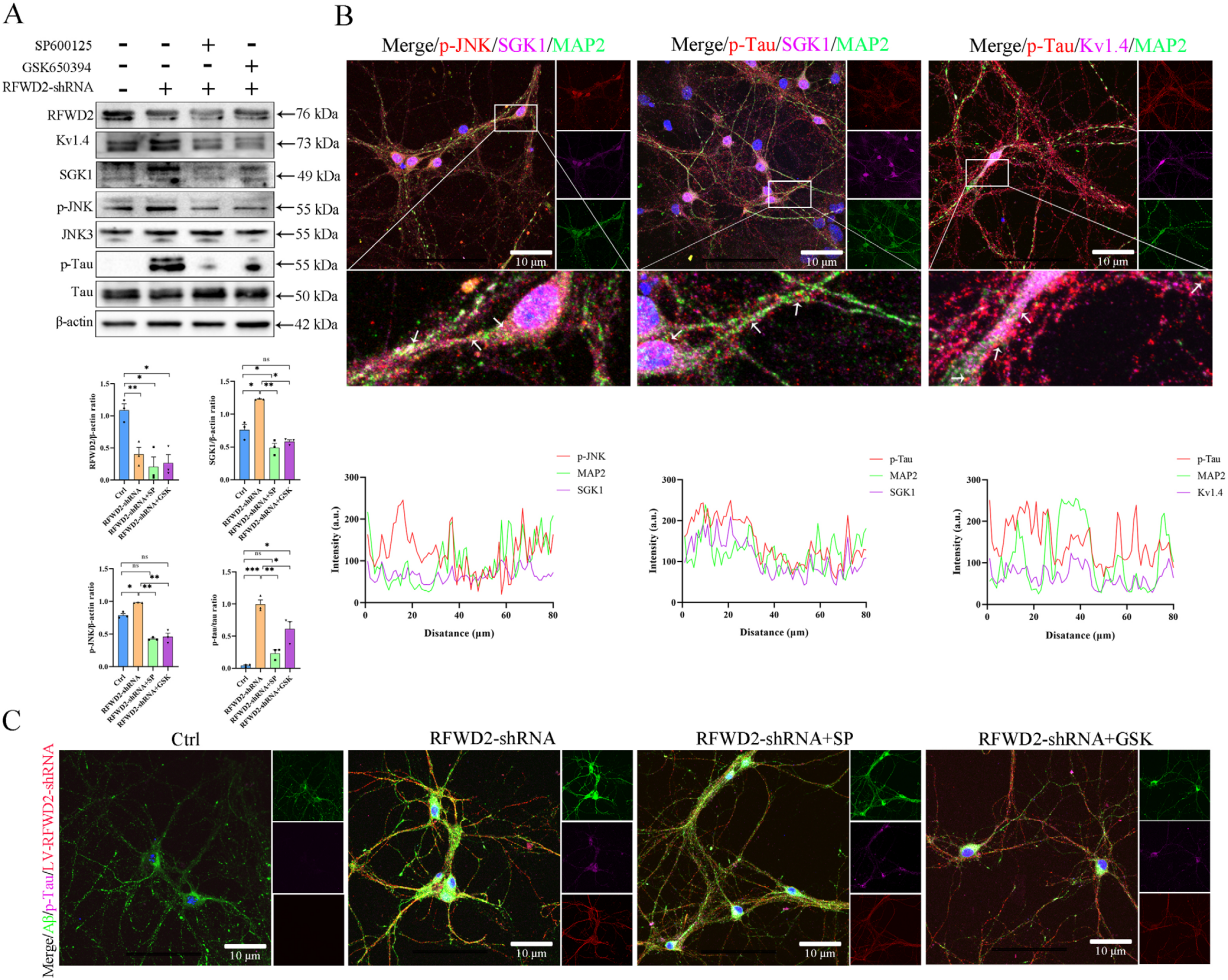
Effects of the RFWD2–JNK–SGK1 axis on the expression and distribution of related proteins in cortical neurons. (A) Western blotting results of related proteins in cortical neurons; the bottom panel shows the gray value analysis of three repeats. (B) Fluorescence colocalization detection of related proteins in cortical neurons. Green fluorescence represents MAP2, and blue fluorescence represents DAPI. Left: Red, *p*‐JNK; purple, SGK1. Middle: Red, p‐Tau; purple, SGK1. Right: Red, p‐Tau; purple, Kv1.4. The Pearson correlation coefficient analysis results are shown at the bottom. (C) Changes in the protein expression levels of p‐Tau and Aβ in cortical neurons from different treatment groups. Green, Aβ; red, LV‐RFWD2‐shRNA; purple, p‐Tau.

Inhibition of RFWD2 expression activated the JNK pathway and caused significant increases in the expression levels of SGK1 and p‐Tau (Figure 6A). After treatment with SP600125 and GSK650394, these abnormalities significantly improved. These results indicate that the RFWD2–JNK–SGK1 axis regulates changes in p‐Tau expression levels (Figure 6A). Moreover, *p*‐JNK and SGK1 colocalized in the axons and soma of neurons, whereas SGK1 and p‐Tau mainly colocalized in the axons. In addition, *p*‐Tau and Kv1.4 colocalized in the axons and soma (Figure 6B).

Inhibiting the expression of RFWD2 also increased Aβ and p‐Tau levels, and this increasing trend was significantly reduced after JNK and SGK1 inhibition (Figure 6C). These results indicate that the RFWD2–JNK–SGK1 axis can regulate the expression of *p*‐Tau and Aβ.

### 3.7 Effects of the RFWD2–JNK–SGK1 Axis on Mitochondrial Function and Apoptosis in Cortical Neurons

**FIGURE 7:**
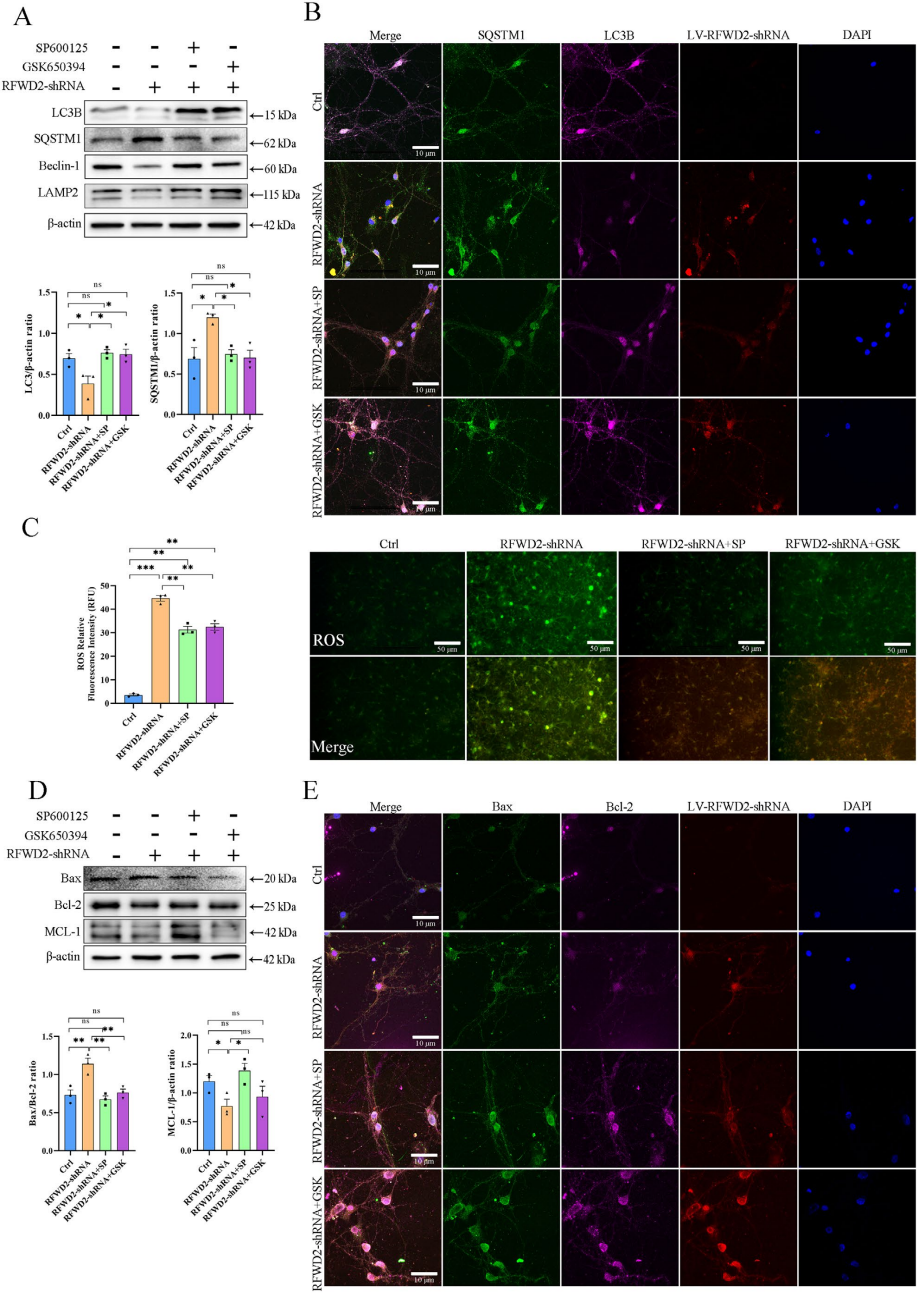
(A) Western blotting analysis of autophagy‐related proteins in cortical neurons. The bottom panel shows the gray value analysis of three repeats. (B) Immunofluorescence detection of autophagy‐related proteins in cortical neurons. Green, SQSTM1; purple, LC3; red, LV‐RFWD2‐shRNA; blue, DAPI. (C) Detection of ROS production in cortical neurons from different treatment groups. (D) Western blotting and gray value analyses of the expression of apoptosis‐related proteins (Bax, Bcl‐2, and MCL‐1). (E) Immunofluorescence detection of apoptotic proteins. Green, Bax; purple, Bcl‐2; red, LV‐RFWD2‐shRNA; blue, DAPI.

Downregulation of RFWD2 expression resulted in a significant decrease in expression levels of the mitochondrial autophagy proteins, LC3 and Beclin‐1, and lysosome‐associated membrane protein 2 (LAMP2), whereas expression of the autophagy substrate protein, SQSTM1, significantly increased (Figure 7A). However, these abnormalities improved after treatment with SP600125 and GSK650394. The immunofluorescence results were highly consistent with the western blotting results (Figure 7B). Moreover, ROS production significantly increased in the RFWD2‐shRNA group, indicating a high level of oxidative stress (Figure 7C). Contrastingly, ROS levels in the RFWD2‐shRNA + SP and RFWD2‐shRNA + GSK groups were significantly lower. In addition, the Bax/Bcl‐2 ratio significantly increased in the RFWD2‐shRNA group, and expression levels of the antiapoptotic protein, MCL‐1, significantly decreased (Figure 7D) These results indicate that the downregulation of RFWD2 expression caused mitochondrial autophagy dysfunction in neurons, inhibited lysosome‐related functions, sharply increased oxidative stress levels, and induced apoptotic signals within cells. However, these phenomena were significantly diminished after inhibition of the JNK pathway and when SGK1 protein levels were reduced.

### 3.8 Inhibition of JNK and SGK1 Expression Can Alleviate Cognitive Impairment in Mice With RFWD2 Expression

**FIGURE 8:**
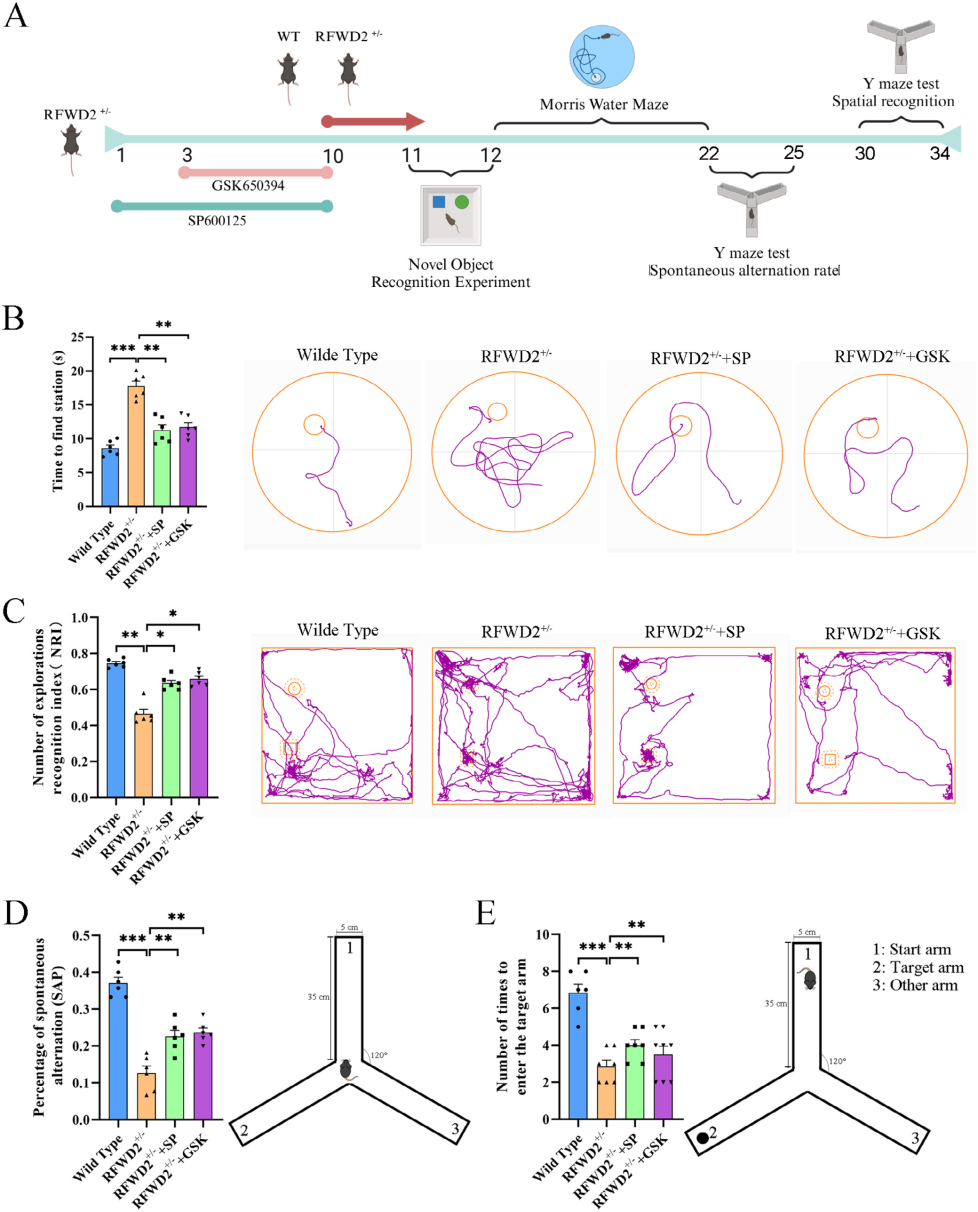
Behavioral effects of the RFWD2–JNK–SGK1 axis on novel object recognition, water maze spatial memory, and Y maze working memory in mice. (A) Timeline of the intraperitoneal injection of drugs in mice and behavioral tests. The RFWD2^+/−^ + SP600125 group received an intraperitoneal injection of SP600125 on days 1–10; the RFWD2^+/−^ + GSK650394 group received an intraperitoneal injection of GSK650394 on days 3–10; and the Wild‐Type and RFWD2^+/−^ groups received no treatment. On the 10th day, behavioral tests were simultaneously conducted for all four groups. (B) Novel object recognition behavioral test. The horizontal coordinate of the bar graph represents mice in different treatment groups, and the vertical coordinate represents the platform‐finding time. The right side shows the platform‐finding trajectories of mice during the water maze test. (C) The horizontal coordinate of the bar graph represents mice in different treatment groups, and the vertical coordinate represents the cognitive index (number of explorations of the novel object/number of explorations of the novel object + old object). The right side shows the trajectory during exploration of the novel and old objects. (D) Y‐maze test. The vertical coordinate represents the spontaneous alternation rate of mice exploring each arm in the Y maze (number of consecutive entries into three arms/total number of arm entries). (E) Statistical analysis of the number of entries of mice into the target arm.

SP600125 (10 mg/kg, days 1–10) and GSK650394 (30 mg/kg, days 3–10) were injected into mice of the RFWD2^+/−^ group to inhibit the expression of JNK and SGK1, respectively (Figure 8A), whereafter behavioral tests were conducted. In the water maze test, the platform‐finding time of mice in the RFWD2^+/−^ group was significantly longer than that of mice in the Wild‐Type group (*p* < 0.01, *n* = 10; Figure 8B). Mice in this group performed poorly in terms of spatial learning and memory abilities and had difficulty finding the platform hidden in the water quickly and accurately. However, the platform‐finding times of mice in the RFWD2^+/−^ + SP and RFWD2^+/−^ + GSK groups were significantly shorter than those in the RFWD2^+/−^ group.

In the novel object recognition test (Figure 8C), results revealed that both the exploration (*p* < 0.05, *n* = 10) and exploration times (*p* < 0.001, *n* = 10) of mice in the RFWD2^+/−^ group for the new object decreased, and that their cognitive index (NRI) also significantly decreased. Compared with those in the RFWD2^+/−^ group, the number of new objects recognized by mice in the RFWD2^+/−^ group increased, and their cognitive index improved. These findings indicate that inhibition of the JNK and SGK1 signaling pathways can improve cognitive dysfunction caused by the inhibited expression of RFWD2 to a certain extent.

In the Y‐maze test, the SAP value of mice in the RFWD2^+/−^ group significantly decreased by 24.45% (*p* < 0.05, *n* = 10) (Figure 8D), and the number of entries into the target arm during the spatial recognition memory test decreased from 6 to 8 to 2–3 times (Figure 8D). However, SAP values of the RFWD2^+/−^ + SP and RFWD2^+/−^ + GSK groups decreased by 14.53% and 13.38%, respectively, compared with those of the Wild‐Type group; the SPA values, however, increased by 9.9% and 11.06%, respectively, when compared with those of the RFWD2^+/−^ group (Figure 8D), and the number of entries into the target arm increased to 4–5 times (Figure 8E). These findings indicate that inhibiting the JNK pathway and SGK1 protein expression can alleviate cognitive dysfunction caused by a decrease in RFWD2 expression and restore the working memory weakened by inhibited RFWD2 expression.

### 3.9 RFWD2–JNK–SGK1 Axis Mediates Autophagy and Apoptosis Processes in Mouse Brain Tissue

**FIGURE 9:**
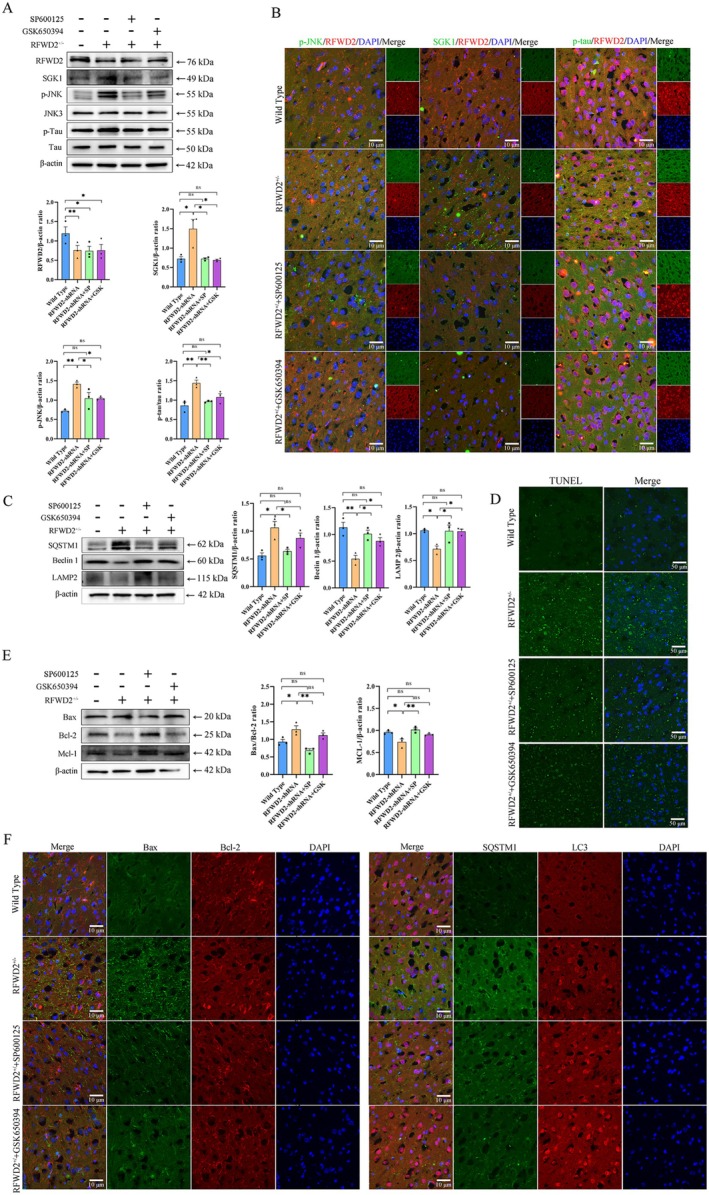
The RFWD2–JNK–SGK1 axis mediates autophagy and apoptosis in mouse brain tissue. (A) The expression of key proteins in brain tissue was detected via western blotting. The bottom panel shows the bar graph analysis of the gray values. (B) Immunofluorescence double‐staining results. Green represents the target proteins (*p*‐JNK, SGK1, and *p*‐Tau); red represents RFWD2; and blue represents DAPI. (C) Western blotting detection of autophagy‐related proteins (LAMP2, Beclin 1, etc.). (D) TUNEL staining of brain tissue; green indicates TUNEL, blue indicates DAPI. (E) Western blotting detection of changes in the expression of apoptotic, antiapoptotic (Bcl‐2 and MCL‐1), and proapoptotic (Bax) proteins. On the right is the gray value analysis of three repetitions. (F) Immunofluorescence double‐staining was used to detect the expression levels of Bax, Bcl‐2, SQSTM1, and LC3 in mouse brain tissue.

The expression levels of SGK1, p‐JNK, and p‐Tau in the cerebral cortex of RFWD2^+/−−^ knockout mice increased, whereas this was prevented after the intraperitoneal injection of SP600125 or GSK650394 (Figure 9A). The immunohistochemical results revealed that the expression levels of *p*‐JNK, SGK1, and *p*‐Tau were consistent with the western blotting results (Figure 9B). Moreover, RFWD2 and *p*‐JNK were specifically colocalized in neurons of the brain tissue, and interactions between RFWD2, *p*‐Tau, and SGK1 were also observed (Figure 9B). In addition, the expression levels of Beclin 1, Bcl‐2, and MCL‐1 decreased, whereas those of SQSTM1 increased (Figure 9C,D). The immunofluorescence results were consistent with these findings (Figure 9F), which supported that abnormalities in autophagic flux occurred at the tissue level and the expression balance of proapoptotic and antiapoptotic proteins was disrupted. Finally, TUNEL staining revealed that the neurons in tissues of the RFWD2^+/−^ group underwent apoptosis, and the degree of apoptosis was lower in the RFWD2^+/−^ + SP600125 and RFWD2^+/−^ + GSK650394 groups. In summary, the abnormal autophagy and increased apoptosis caused by the RFWD2–JNK–SGK1 axis at the tissue level were consistent with results obtained at the PC12 and cortical neuron levels, and this axis jointly regulates brain function.

## 4 Discussion

### 4.1 Analysis of the Multidimensional Role of RFWD2 in the Pathogenesis of AD

AD is a neurodegenerative disease that still causes fear among many patients and seriously affects their quality of life. It manifests at the clinical symptom level as decreased cognitive, learning, and memory abilities [23]. At the molecular level, excessive phosphorylation of the Tau protein constitutes the core feature of neuronal lesions in the brain [24]. RFWD2‐mediated oxidative stress and neuroinflammation continuously influence the progression of AD; however, the development of numerous drugs has led to only a slight alleviation [25]. Research by Cao et al. revealed that inhibiting histone H3K4‐specific methyltransferase can improve the cognitive and synaptic functions in AD mouse models. Their research emphasized the significance of epigenetic regulation and highlighted the key role of *SGK1* in AD [26]. Similarly, by comparing the hippocampal transcriptome analysis of sleep deprivation and AD mouse models, Wei discovered DEGs shared between the two and explored the potential connection between them and the development of AD [27]. These findings suggest a highly promising new target for therapeutic intervention in AD.

In the present study, we analyzed transcriptome sequencing results and found that in vivo RFWD2 knockout also caused differential expression of the *SGK1* gene. Further exploration of the connection between RFWD2 and these processes revealed the co‐pathogenic mechanism of the RFWD2–JNK–SGK1 axis in AD. In‐depth analysis of DEGs in the AD database revealed that *SGK1*, *Card9*, *Ptpn6*, *Fgg*, and *Fga* are closely associated with the MAPK pathway. The activation of MAPK signaling can promote mitochondrial fragmentation and activate glycolysis to replace mitochondrial oxidative metabolism [28]. Research has shown that RFWD2 plays a non‐negligible role in regulating mitochondrial function [29]. Therefore, we speculated that changes in DEGs caused by the reduction in RFWD2 through interaction with the MAPK pathway are deeply involved in the pathogenesis of AD. These findings provide a new perspective for understanding the pathogenesis of abnormal protein aggregation in AD.

### 4.2 Colocalization and Interaction of p‐JNK, SGK1, and RFWD2 in Neurons

JNK belongs to a subfamily of MAPK members and transactivates the substrate, c‐Jun, by phosphorylating the N‐terminal Ser63 and Ser73 residues [30]. Once JNK is activated into p‐JNK, it phosphorylates nearly 100 protein substrates. Growth‐associated protein 43 is a marker of axonal growth and regeneration that acts through the phosphorylation of JNK [31]. Previous studies have shown that SGK1 can influence the transmembrane transport of ions by regulating the probability of channel openings or deactivations [32]. Additionally, some studies have indicated that activation of the JNK signaling pathway can relieve the partial inhibitory effect on SGK1 expression and play a role in the formation of long‐term memory [17]. However, the regulatory relationship between RFWD2 and SGK1 has yet to be clarified. In the present study, we revealed interactions among RFWD2, SGK1, and *p*‐JNK and found that their co‐localization in cortical neurons indicates the existence of direct or indirect interactions. The phosphorylation status of proteins related to neurotransmitter release may be regulated by *p*‐JNK, such as regulating the function of synaptic vesicle‐related proteins, whereas RFWD2 and SGK1 may jointly participate in the complex regulatory network of neurotransmitter release through the regulation of p‐JNK and indirect influence on ion channels. Under the pathological conditions of AD, an imbalance in this regulatory network may be an important molecular mechanism leading to dysfunction of the neurotransmitter system.

### 4.3 Discussion of the Synergistic Pathogenic Mechanism of the RFWD2–JNK–SGK1 Axis in AD

The development of AD is closely associated with mitochondrial dysfunction. Abnormal activation of the JNK signaling pathway can upregulate the expression of mitochondrial fission proteins, such as dynamin‐related protein 1, thereby leading to fragmentation of the mitochondrial network and abnormal morphology [33]. The abnormal activation of SGK1 can further interfere with the functions of mitochondrial metabolic enzymes or transporter proteins, exacerbating the impairment of mitochondrial oxidative phosphorylation function [34]. The present study revealed that a reduction in RFWD2 caused a significant increase in expression levels of the JNK signaling pathway and SGK1, whereas those of LC3B, Beclin 1, and SQSTM1 decreased. These abnormal manifestations improved after treatment with JNK and SGK1 inhibitors. These findings suggest that the RFWD2–JNK–SGK1 axis not only indirectly influences the morphological structure of mitochondria but can also lead to autophagy dysfunction. In addition, ROS generation is regulated by the RFWD2–JNK–SGK1 axis. SGK1 abnormalities may further weaken the ability of the intracellular antioxidant defense system to scavenge ROS. Additionally, the expression levels of LAMP2 were significantly increased. Given that LAMP2 is an important protein in the lysosomal membrane and closely related to lysosomal function and autophagy, this phenomenon suggests that the RFWD2–JNK–SGK1 axis also plays a regulatory role in autophagy that involves lysosomes. However, the specific mechanism and impact of this regulatory role require further in‐depth studies.

In AD, Aβ activates the JNK signaling pathway, which not only damages mitochondrial function in neurons but also triggers neuronal apoptosis [35]. The present study showed that RFWD2 led to an abnormal increase in the Bax/Bcl‐2 ratio, and treatment with GSK650394 and SP600125 could, to a certain extent, improve the phosphorylation levels of Tau protein caused by the decrease in RFWD2 and increase in expression of the antiapoptotic protein, MCL‐1. These findings further indicate that the RFWD2–JNK–SGK1 axis has a close synergistic relationship with AD‐related apoptotic processes. The abnormal expression of RFWD2 may indirectly regulate the expression of apoptosis‐related proteins by influencing the activities of JNK and SGK1, thereby affecting neuronal survival. As an upstream regulator, RFWD2 integrates JNK and SGK1 to form an axial regulatory mechanism, which has a unique significance in AD apoptosis.

### 4.4 Identification of the Upstream and Downstream Relationships Between JNK and SGK1

Under normal physiological conditions, RFWD2 maintains a relatively stable molecular balance within cells at a relatively stable level [36]. In specific brain regions or cell models of patients with AD, the expression levels of RFWD2 significantly fluctuate, which may stem from various stimuli, such as oxidative stress and abnormal protein aggregation [25]. When the expression of RFWD2 decreases, the JNK signaling pathway is abnormally activated, suggesting that RFWD2 may have a negative regulatory effect on JNK. Under normal circumstances, RFWD2 inhibits the excessive activation of JNK, and its functional impairment thus leads to uncontrolled JNK activity. JNK is an intermediate key link in this axis after activation, and changes in its activity significantly regulate downstream SGK1 expression. The regulation of SGK1 by abnormally activated JNK may cause SGK1 to deviate from its normal functional range [37]. The present study revealed that after the abnormal activation of JNK caused by a change in RFWD2 expression, the expression levels and activity of SGK1 changed accordingly, indicating that JNK has a positive regulatory effect on SGK1. Therefore, RFWD2 may play a key initiating role in the upstream regulation of the RFWD2–JNK–SGK1 axis.

SGK1 is a downstream molecule of the RFWD2–JNK–SGK1 axis, and its abnormal expression and activity may affect the functions of upstream molecules. SGK1 can indirectly affect the functions of RFWD2 and JNK by regulating intracellular ion balance, metabolic processes, and interactions with other signaling pathways [38]. SGK1 may affect intracellular energy metabolism, which is related to the regulation of RFWD2 expression. In a cellular environment with insufficient energy, changes in SGK1 activity can affect the transmission of certain transcription factors or signaling molecules [39]; therefore, this feedback is speculated to regulate the expression levels of RFWD2. SGK1 affects the activity of antioxidant enzymes, causing excessive intracellular ROS accumulation, which can activate the JNK signaling pathway, forming a complex feedback loop [40, 41]. In AD, this feedback loop may continuously amplify pathological signals and accelerate neuronal damage and death.

Along the entire RFWD2–JNK–SGK1 axis, the upstream and downstream regulation between molecules does not exhibit a simple linear relationship but rather involves a complex mechanism of crosstalk and feedback regulation. In addition to being regulated by RFWD2 and acting on SGK1, JNK may be feedback‐regulated by SGK1. This complex network plays a collaborative role in AD‐related neuronal dysfunction and other pathological processes. In neurons, abnormally activated JNK and SGK1 are transmitted upstream and downstream of this axis and can affect multiple key processes, such as mitochondrial function, apoptosis, and synaptic plasticity. Abnormal activation of the JNK signaling pathway can upregulate the expression of mitochondrial fission proteins, leading to breakdown of the mitochondrial network. Abnormal activation of SGK1 can further interfere with the function of mitochondrial metabolic enzymes and aggravate mitochondrial damage. The synergy between these two components accelerates the imbalance of neuronal energy metabolism and functional decline. Moreover, in terms of apoptosis, JNK can regulate apoptosis‐related proteins, and SGK1 can jointly promote neuronal apoptosis by affecting ion balance and energy metabolism, ultimately promoting the progression of AD. Through in vivo and in vitro experiments, the present study analyzed the synergistic pathogenesis of the RFWD2–JNK–SGK1 axis, providing a more systematic and comprehensive perspective for furthering our understanding of AD pathogenesis. Identifying precise therapeutic targets and developing more effective AD intervention strategies would be helpful in future studies.

### 4.5 Reflection on Limitations: Current Dilemmas and Future Exploration of RFWD2 Research

Although this study revealed the effect that RFWD2 decline has on JNK pathway activation and related protein expression, some limitations need to be addressed. First, in the evaluation of animal models, this study mainly used new and old object recognition, water maze, and Y‐maze experiments to explore the effects that the RFWD2–JNK–SGK1 axis has on the cognitive function and spatial memory of mice, and thereafter inferred whether AD‐like symptoms had occurred. However, this method is a relatively simple way to judge AD symptoms. Although we referenced the AD mouse model database for comparison, future studies need to include more comprehensive indicators and add studies related to human patient brain tissue for a comprehensive evaluation to more accurately determine the effect that the RFWD2–JNK–SGK1 axis has on the development of AD‐like symptoms. Second, in terms of drug recovery trials, most AD therapies currently on the market only relieve symptoms and lack specificity, greatly limiting our ability to conduct relevant trials. This not only prevents us from deeply exploring the efficacy of RFWD2 in drug intervention and recovery but also from fully evaluating its potential value and practical application prospects in the treatment of AD. In addition, the main goal of this study was to preliminarily explore the relationship between RFWD2 and the JNK pathway, SGK1, and Tau proteins. The scope and depth of the study had to be balanced with the limited resources and time available. Therefore, this study could not clarify whether RFWD2 regulates JNK activity through specific molecular mechanisms, such as ubiquitination. Future studies should further explore the detailed molecular mechanisms underlying this regulatory process, which will contribute to a more comprehensive understanding of the mechanism of action of the RFWD2–JNK pathway and its significance in related physiological and pathological processes.

## 5 Conclusion

Our study focused on the key process by which RFWD2 affects SGK1 expression via the JNK pathway. The RFWD2–JNK–SGK1 axis regulates the cognitive and memory functions of mice, mainly by mediating mitochondrial functions and apoptosis, affecting the phosphorylation status of the Tau protein, and participating in the occurrence and development of AD. In addition, the RFWD2–JNK–SGK1 axis exerts a certain degree of influence on potassium ion channels. Overall, this study provides valuable directions and a foundation for in‐depth exploration of the mechanism of action of the RFWD2–JNK–SGK1 axis in the occurrence and development of AD and subsequent related research.

Guidelines for the Ethical Review of Laboratory Animal Welfare (GB/T 35892‐2018), and the ethics of laboratory animal welfare were standardized. All experiments were designed and reported in accordance with the Animal Studies: Reporting in vivo experiments (ARRIVE) guidelines. The animal facilities and protocols have complied with the Guide for the Care and Use of Laboratory Animals (USA National Research Council, 1996). The use of laboratory animals and all experimental procedures were approved by the Institutional Animal Care and Use Committee (IACUC) for Ethics of Bengbu Medical University (approval no. 2024–565).

## Author Contributions

Mengjiao Ying designed and conceived the study. Mengjiao Ying, Xiaochuan Qi, and Ao Wang performed all experiments and carried out data analysis. Mengjiao Ying, Xiaochuan Qi, Ao Wang, Wenhui Tong, Danting Yu, and Guangshang Zhong participated in data interpretation and contributed to analysis of the results. Gaofeng Liu and Yu Guo contributed to the inference and identification of Alzheimer's disease risk genes using NCBI data resources. Mengjiao Ying, Ao Wang, and Wenhui Meng drafted the manuscript. Gaofeng Liu and Yu Guo critically revised the manuscript for important intellectual content and supervised the overall study. All authors read, reviewed, and approved the final version of the manuscript prior to submission.

## Funding

This work was supported by National Natural Science Foundation of China, 82371382, 81771381. Natural Science Foundation of Anhui Province, 2308085MH256. Science Research Project of Bengbu Medical University, 2021byfy002. Postgraduate Innovative Training Program of Bengbu Medical University, Byycx23007. Undergraduate Innovative Training Program of China, 202410367024.

## Consent

The authors have nothing to report.

## Conflicts of Interest

The authors declare no conflicts of interest.

## Supporting information


**Figure S1:** Validation of RFWD2‐knockdown PC12 cell models via mRNA/protein detection and grayscale analysis of Western Blot results.
**Figure S2:** Concentration‐dependent analysis of SP600125/GSK650394 in PC12 cells (CCK8 viability assay, Western Blot detection and grayscale statistics of p‐JNK/SGK1).
**Figure S3:** BioRender academic license for the manuscript's Graphical Abstract
**Figure S4:** BioRender official license for the behavioral test flowchart in the main manuscript's Figure 8.
**Figure S5:** RFWD2 expression detection in mCherry‐labeled lentivirus‐transfected cells (Western Blot and grayscale quantitative analysis).
**Figure S6:** Bubble plots of pathway clustering analysis for differential gene expression (W‐A/W‐R/A‐R groups), with JNK pathway‐related pathways marked in red.
**Figure S7:** Mechanistic analysis of RFWD2‐JNK interaction (Western Blot detection of p‐JNK/RFWD2 in APP/PS1 model; JNK ubiquitination assay via WB in RFWD2‐knockdown cells with MG132 treatment).

## Data Availability

The datasets generated and/or analysed during the current study are not publicly available due personal privacy protection but are available from the corresponding author on reasonable request.
