# Supplementary material for: RFWD2 Mitigates AD‐Like Cognitive Impairments via the JNK–SGK1 Signaling Pathway in Mice
Source: CNS Neurosci Ther. 2026 Apr 9;32(4):e70860. doi: 10.1002/cns.70860 (PMC13064414; doi:10.1002/cns.70860)
Supplement: Supplementary file 1 — Figure S1: Validation of RFWD2‐knockdown PC12 cell models via mRNA/protein detection and grayscale analysis of Western Blot results. Figure S2: Concentration‐dependent analysis of SP600125/GSK650394 in PC12 cells (CCK8 viability assay, Western Blot detection and grayscale statistics of p‐JNK/SGK1). Figure S3: BioRender academic license for the manuscript's Graphical Abstract Figure S4: BioRender official license for the behavioral test flowchart in the main manuscript's Figure 8. Figure S5: RFWD2 expression detection in mCherry‐labeled lentivirus‐transfected cells (Western Blot and grayscale quantitative analysis). Figure S6: Bubble plots of pathway clustering analysis for differential gene expression (W‐A/W‐R/A‐R groups), with JNK pathway‐related pathways marked in red. Figure S7: Mechanistic analysis of RFWD2‐JNK interaction (Western Blot detection of p‐JNK/RFWD2 in APP/PS1 model; JNK ubiquitination assay via WB in RFWD2‐knockdown cells with MG132 treatment). [file CNS-32-e70860-s001.docx]

**Supplement data**


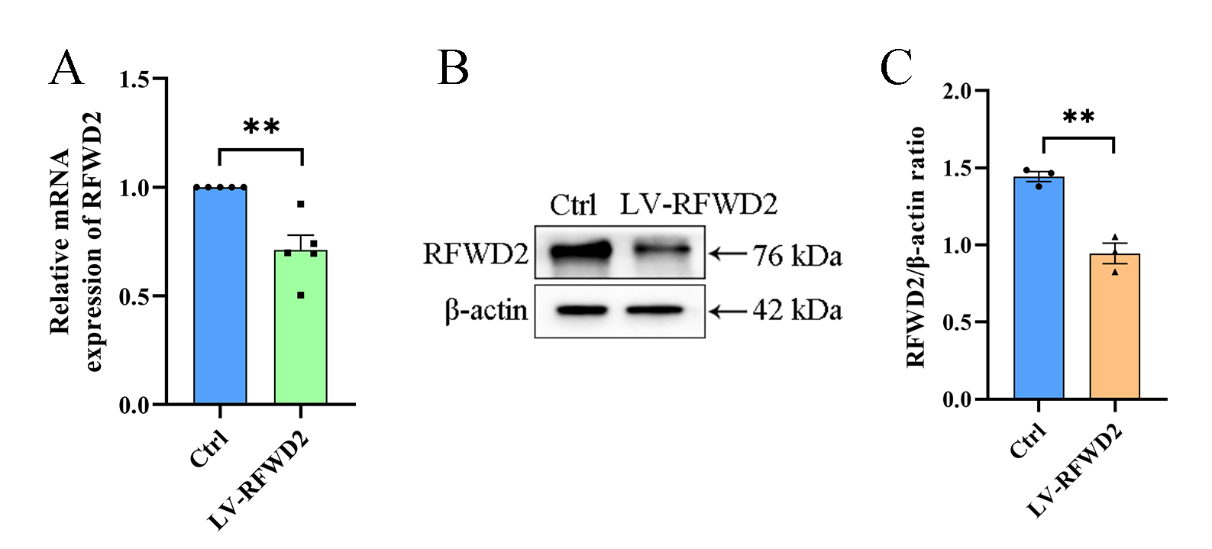


Figure S1. Suggested detection of RFWD2 knockdown cell lines. A. mRNA detection of PC12 transfected with lentivirus for RFWD2 expression inhibition; B. WB detection of PC12 transfected with lentivirus for RFWD2 expression inhibition; C. Gray value analysis of RFWD2 expression level in Western Blot results.


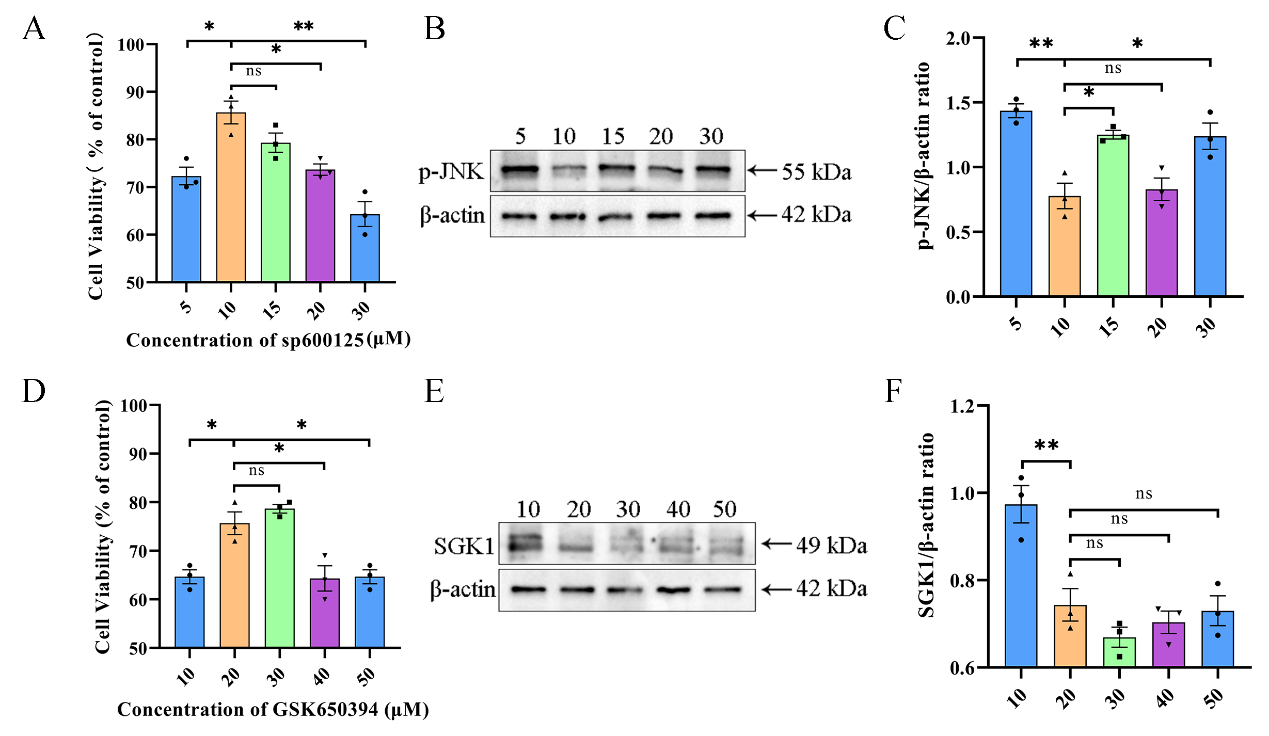


Figure S2. Concentration-dependent analysis of SP600125 and GSK650394. A. CCK8 assay of PC12 cells treated with SP600125 at concentrations of 5, 10, 15, 20, and 30 μM; B. Western blot (Western Blot) analysis after treatment with different concentrations of SP600125; C. Grayscale value statistics of p-JNK expression in Western Blot results; D. CCK8 assay of PC12 cells treated with GSK650394 at concentrations of 10, 20, 30, 40, and 50 μM; E. Western Blot analysis after treatment with different concentrations of GSK650394; F. Grayscale value statistics of SGK1 expression in Western Blot results.


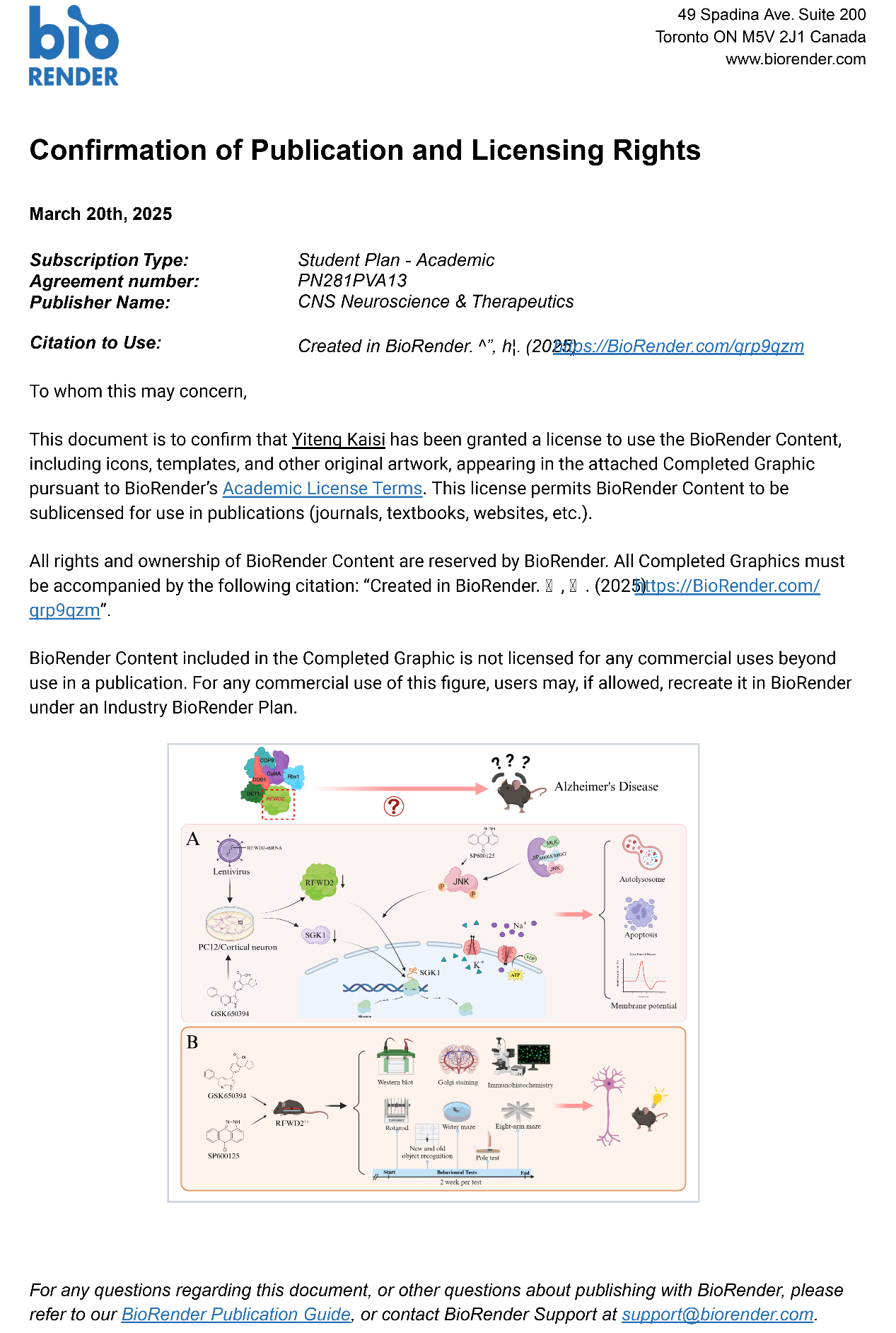


Figure S3. License for Graphical Abstract


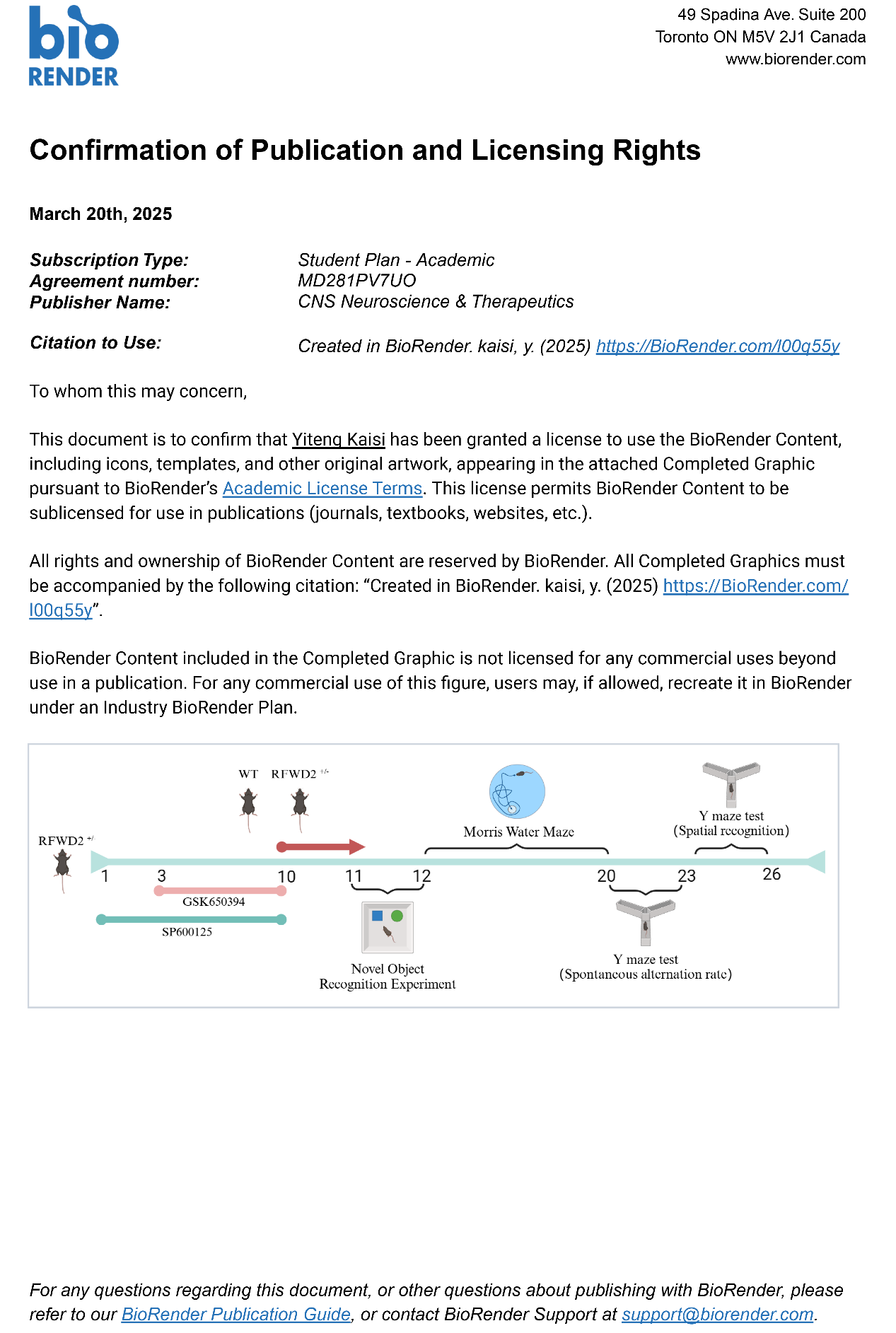


Figure S4. the license for the flowchart of behavioral tests in Figure 8.


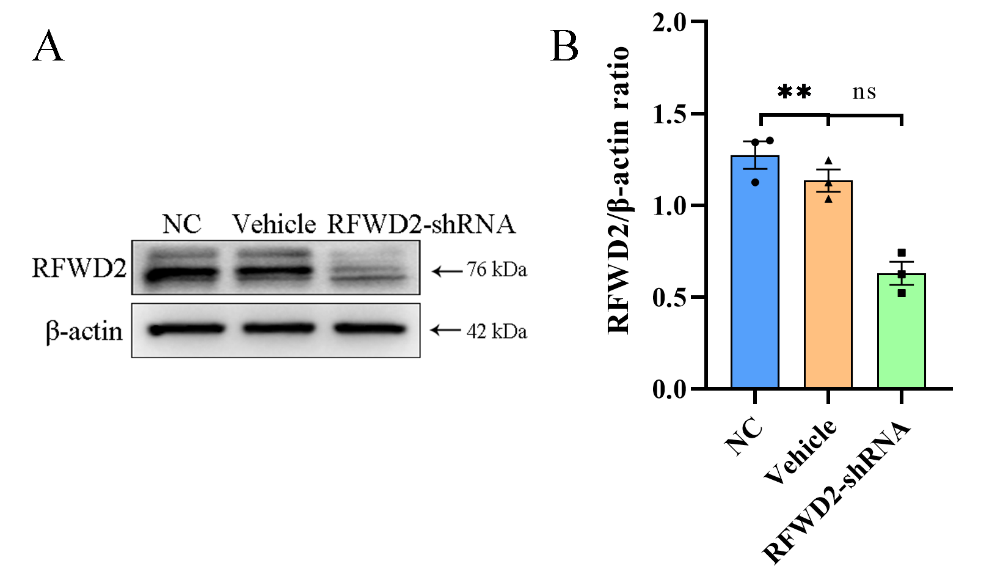


Figure S5. Detection of RFWD2 expression level after lentivirus transfection with mCherry into cells. A. NC - blank control group, Vehicle - empty virus group with mCherry, RFWD2 - shRNA - virus group with mCherry for inhibiting RFWD2 expression. B. Gray value analysis of RFWD2 expression level in Western Blot results。


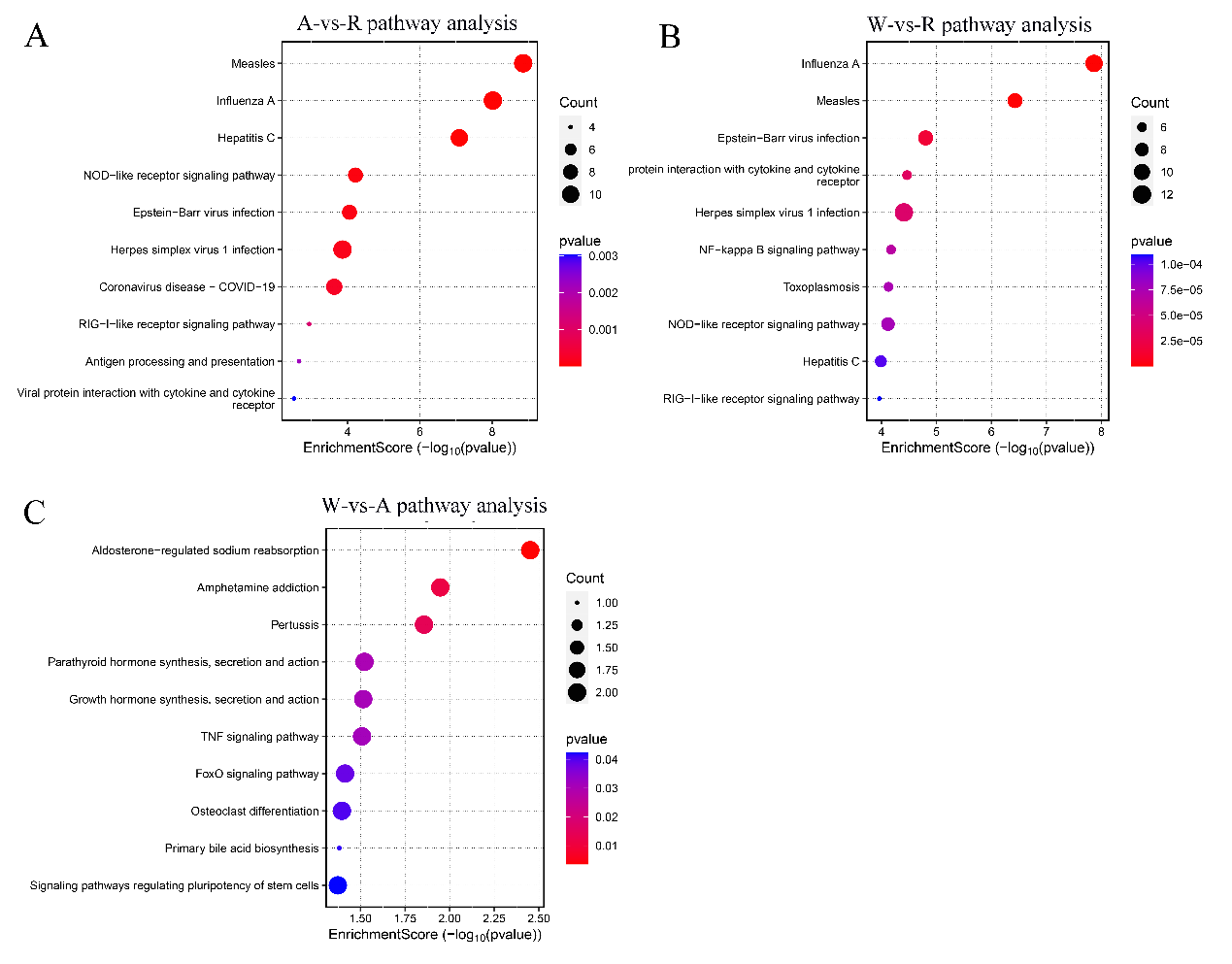


Figure S6: A-C. Bubble plot of pathway clustering analysis of differential gene expression heatmaps of the three groups W-A, W-R, and A-R, with the JNK pathway-related pathways circled in the red box


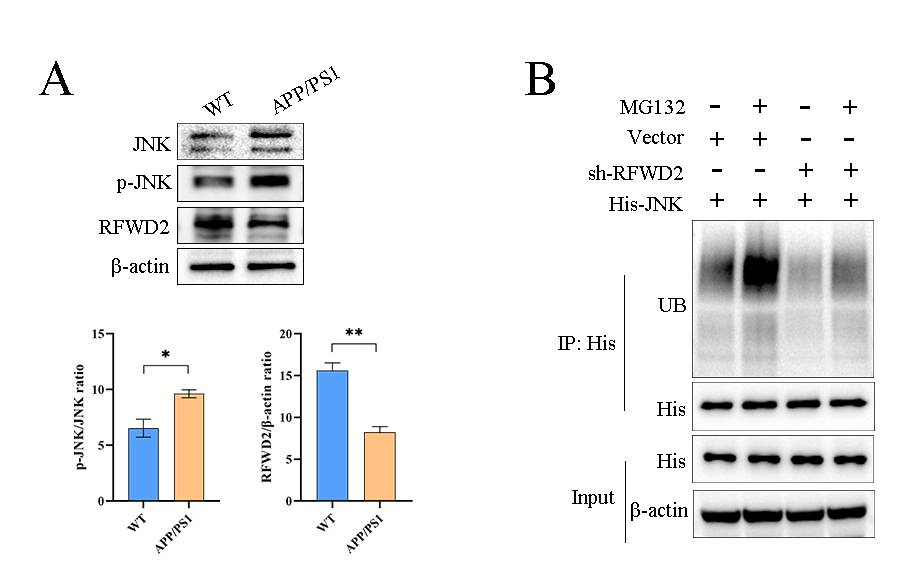


Figure S7: A. The above figure shows the changes in JNK phosphorylation and RFWD2 expression in the APP/PS1 model detected by Western blo, and the lower figure shows the quantitative statistics of gray values. B. In cells overexpressing His-JNK, empty vectors or RFWD2 knockdown plasmids (sh-RFWD2) were transfected respectively, and the proteasome was inhibited by MG132. The ubiquitination level of JNK was detected by Western Blot.
